# Supplementary material for: Economic evaluation of surgical treatments for women with stress urinary incontinence: a cost-utility and value of information analysis
Source: BMJ Open. 2020 Jun 11;10(6):e035555. doi: 10.1136/bmjopen-2019-035555 (PMC7295417; doi:10.1136/bmjopen-2019-035555)
Supplement: Supplementary data [file bmjopen-2019-035555supp001.pdf]

Appendices

Table S1 Long-term cure rates after Retro-MUS used for extrapolation

Scenario	Time (years)	Cure rate	Source	Scale parameter (λ)	Shape parameter (γ)
Base case analysis	1	84%	Meta-analysis	0.08799444	0.4932644
	2	78%			
Sensitivity analysis 1	1	85%	(1)	0.1969761	0.12368398
	2	80%			
Sensitivity analysis 2	1	78%	(2)	0.19391258	0.1788069
	13	67.5%			

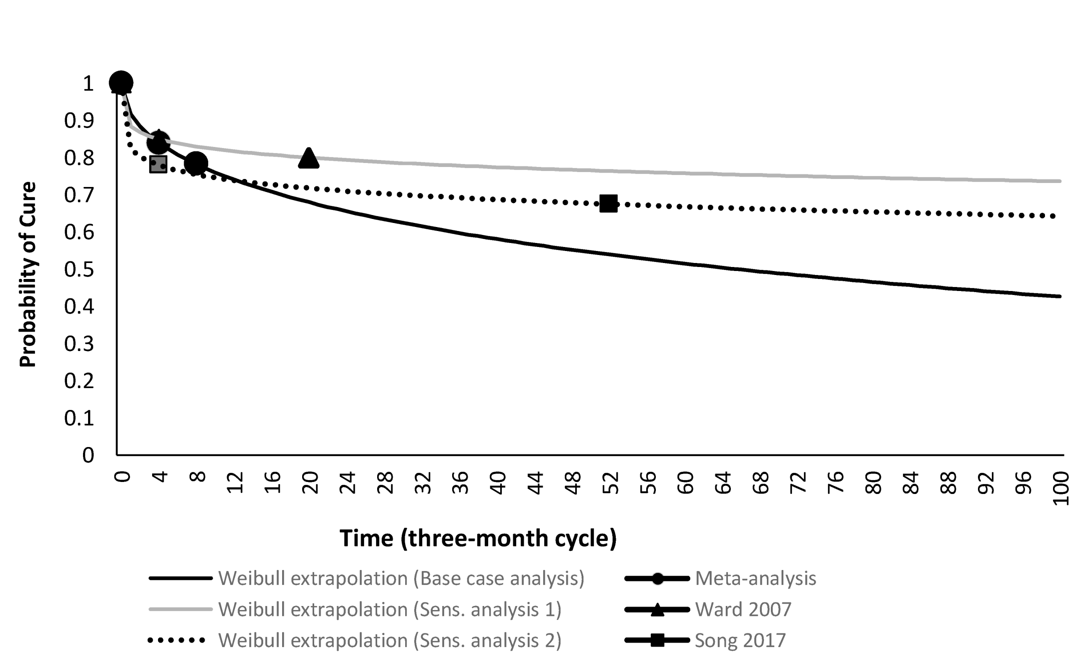

Figure S1 Extrapolated long-term cure rates after Retro-MUS surgery. Note: each cycle is 3 months long

Javanbakht M, *et al. BMJ Open* 2020; 10:e035555. doi: 10.1136/bmjopen-2019-035555

Table S2 Weibull hazard function parameters for estimation of long-term cure rates after Injectable agents

**Weibull Parameters**	**Cure rate**		
	Lower limit	Mean	Upper limit
**Lambda**	1.020173	0.767078	0.548895
**Gamma**	0.120006	0.137820	0.166837

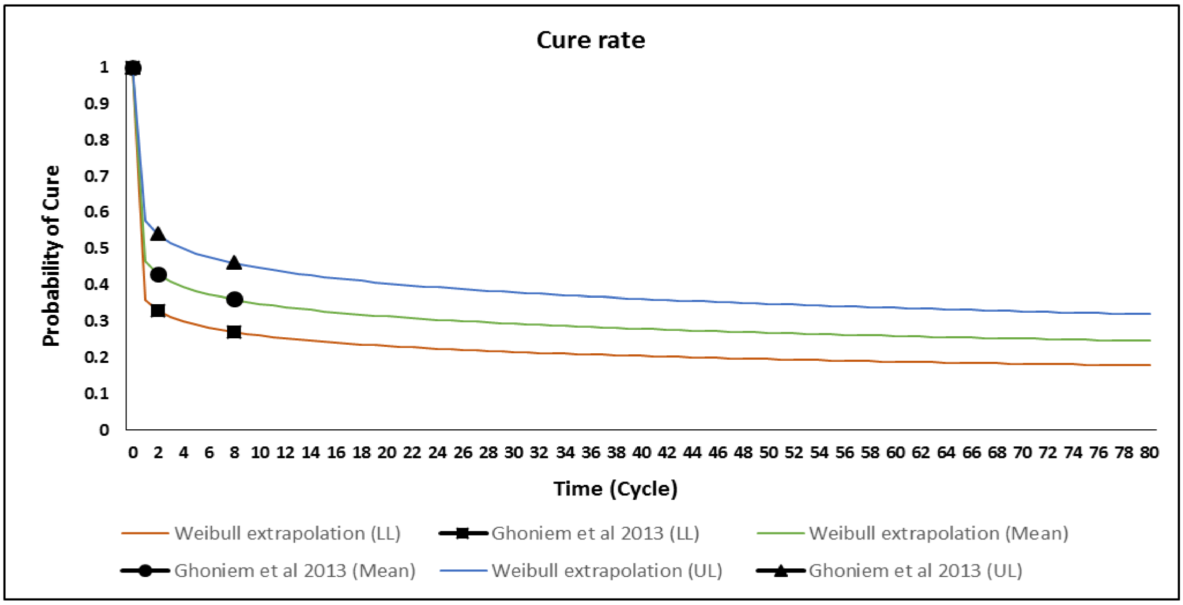

Figure S2 Extrapolated long-term cure rates after peri-urethral injections (Injectable agents). Note that each cycle is 3 months long

Javanbakht M, *et al. BMJ Open* 2020; 10:e035555. doi: 10.1136/bmjopen-2019-035555

Table S3 Complications incidence rates after different surgical treatments (results from random-effect meta-analysis)

**Complication**	**Infection**		**De novo symptoms of urgency or urgency incontinence**		**Voiding difficulties including urinary retention**		**Bladder or urethral perforation**		**Tape/mesh erosion / extrusion/exposure**		**Short term pain**		**Persistent pain or discomfort**	
Surgery	Posterior Mean	Posterior SD	Posterior Mean	Posterior SD	Posterior Mean	Posterior SD	Posterior Mean	Posterior SD	Posterior Mean	Posterior SD	Posterior Mean	Posterior SD	Posterior Mean	Posterior SD
Anterior repair	NA	NA	15.84%	11.83%	0.01%	0.15%	NA	NA	NA	NA	NA	NA	NA	NA
Bladder neck needle	NA	NA	8.14%	4.87%	1.67%	2.61%	NA	NA	NA	NA	57.64%	30.08%	NA	NA
Open-colpo	24.33%	21.82%	5.78%	1.15%	3.00%	1.04%	1.02%	0.50%	0.0%	NA	20.94%	25.65%	0.15%	2.58%
Lap-colpo	NA	NA	8.38%	3.98%	6.32%	2.35%	2.10%	1.22%	0.0%	NA	NA	NA	NA	NA
Trad-sling	14.85%	12.49%	7.85%	3.23%	11.63%	3.60%	1.82%	1.11%	0.17%	0.34%	28.99%	28.12%	7.00%	8.98%
Retro-MUS	3.91%	4.72%	4.30%	0.66%	6.75%	0.95%	4.89%	0.50%	1.40%	0.31%	4.36%	1.14%	5.09%	2.37%
Transob-MUS	2.84%	1.03%	5.31%	0.51%	2.88%	0.44%	5.13%	0.50%	2.08%	0.26%	7.04%	2.08%	4.93%	1.22%
Single incision	3.22%	1.20%	6.47%	0.81%	2.15%	0.45%	0.37%	0.18%	2.38%	0.55%	4.54%	3.21%	1.28%	1.39%
Injectable agents	17.94%	24.59%	17.94%	24.59%	11.47%	20.70%	NA	NA	NA	NA	NA	NA	NA	NA

NA: Not available or applicable; Anterior repair: Anterior vaginal repair (anterior colporrhaphy); Bladder neck needle: Bladder neck needle suspensions; CrI: Creditable interval; Lap-colpo: Laparoscopic retropubic colposuspension; Open-colpo: Open abdominal retropubic colposuspension; Trad-sling: Traditional suburethral retropubic sling procedures; MUS: Mid-urethral sling; Retro-MUS: Retropubic mid-urethral sling; Transob-MUS: Transobturator mid-urethral sling; Single incision: Single incision sling procedures (‘mini-slings

Javanbakht M, *et al. BMJ Open* 2020; 10:e035555. doi: 10.1136/bmjopen-2019-035555

Table S4 Costs inputs

Resource use	Cost (£)	Source	HRG code
**Surgery cost**			
Anterior repair	2,254.72	NHS reference costs 2017/18 (3)	MA04C/D - Intermediate open, lower genital tract procedures-elective inpatient
Bladder neck needle	1,978.94	NHS reference costs 2017/18 (3)	LB26A/B - Intermediate endoscopic, prostate or bladder neck procedures-elective
Open-colpo	4,113.30	NHS reference costs 2017/18 (3)	LB59Z - Major, open or laparoscopic bladder neck procedures-elective inpatient
Lap-colpo	4,113.30	NHS reference costs 2017/18 (3)	LB59Z - Major, open or laparoscopic bladder neck procedures-elective inpatient
Trad-sling	2,131.88	NHS reference costs 2017/18 (3)	LB51A/B - Vaginal tape operations for urinary incontinence-elective inpatient
Retro-MUS	1,550.29	NHS reference costs 2017/18 (3)	LB51A/B - Vaginal tape operations for urinary incontinence-day case
Transob-MUS	1,550.29	NHS reference costs 2017/18 (3)	LB51A/B - Vaginal tape operations for urinary incontinence-day case
Single incision	1,103.53	Boyers et al. 2013 (4)	N/A
Injectable agents	1,930.49	Cody et al. 2003 (5)	N/A
Additional costs associated with surgery			
Incontinence pads	112.68 (3 month cost estimate)	NICE guidance document (6)	N/A
Urodynamic test	164.38	NHS reference costs 2017/18 (3)	N/A
Consultation pre-surgery (20 minute consultation with surgical consultant)	48.78	PSSRU 2016 (7)	N/A
Urine dipstick analysis	4.18	NICE clinical guidelines (6)	N/A
Full blood count	6.69	NICE clinical guidelines (6)	N/A
Paracetamol 500mg (AAH) x 32	0.17 (daily cost based on a	BNF 2016 (8)	N/A
Lactulose 10g/15ml (AAH) x 10	0.53 (daily cost based on a	BNF 2016 (8)	N/A

Javanbakht M, *et al. BMJ Open* 2020; 10:e035555. doi: 10.1136/bmjopen-2019-035555

**Urge incontinence treatment costs**			
Bladder training	103.73 (3 month cost estimate)	Appendices of NICE guidelines (6)	N/A
Band 6 hospital-based physiotherapist	47.72	PSSRU 2016 (7)	N/A
Oxybutynin 5mg (AAH) x 56	0.05 (daily cost based on a	BNF 2016 (8)	N/A
Consultant-led non-admitted follow-up	106.05	NHS reference costs 2017/18 (3)	N/A
Intermediate endoscopic bladder	1,029.78	NHS reference costs 2017/18 (3)	N/A
Botulinum toxin 100 unit powder for	146.57	BNF 2016 (8)	N/A
**Adverse event treatment costs**			
Co-amoxiclav 250/125mg (AAH) x 21	0.29 (daily cost based on a	BNF 2016 (8)	N/A
Attention to suprapubic bladder catheter	130.45	NHS reference costs 2017/18 (3)	N/A
Self-catheterisation	6.58 (daily cost based on 4	NICE guidance document (6)	N/A
Mesh excision or repair	1,396.57	NHS reference costs 2017/18 (3)	N/A
Aspirin 300mg (AAH) x 28	0.42 (daily cost based on a	BNF 2016 (8)	N/A

Javanbakht M, *et al. BMJ Open* 2020; 10:e035555. doi: 10.1136/bmjopen-2019-035555

Table S5 Utility values for different health states and utility decrement associated with complications

**Health status**	**Value**	**Source**	**Duration**
SUI pre-treatment	0.78	(9)	Variable
Mixed pre-treatment	0.78	(9)	Variable
Cured from SUI, and no urge urinary incontinence	0.85	(10)	Variable
Urge urinary incontinence	0.74	(10)	Variable
Pure SUI re-treatment	0.74	(10)	Variable
Mixed re-treatment	0.74	(10)	Variable
SUI failure of treatment (containment)	0.74	(10)	Variable
Mixed failure of treatment (containment)	0.74	(10)	Variable
**Utility decrement values for adverse events**			
Adverse event	**Utility decrement**	**Source**	**Duration**
Infection	- 0.19	(11)	2 weeks
Voiding difficulties (long-term)	- 0.23	(11)	3 months
Bladder/urethral perforation	- 0.08	(11)	2 weeks
Tape/mesh exposure/erosion	- 0.25	(11)	3 months
Short-term pain	-0.25	(11)	2 weeks
Persistent pain	- 0.25	(11)	6 months

Javanbakht M, *et al. BMJ Open* 2020; 10:e035555. doi: 10.1136/bmjopen-2019-035555

Deterministic Sensitivity Analyses Results

Table S6 Results from deterministic sensitivity analyses-applying higher incidence rates of mesh complications after MUS procedures (lifetime time horizon)

Strategy	Cost (£)	Incremental cost (£)	QALY	Incremental QALY	ICER (£) (ΔCost/ΔQALY)	Probability cost-effective for different WTP thresholds (%)	
						£20,000	£30,000
Base-case analysis – estimating the incidence rate of mesh complications after MUS procedures based on the data from trials							
Retro-MUS	8,666		24.005			51.0%	48.0%
Trad-sling	9,071	405	24.014	0.009	45,340	43.0%	45.0%
Transob-MUS	10,174	1,103	23.435	-0.580	Dominated	0%	0%
Single incision sling	10,189	1,118	23.221	-0.793	Dominated	0%	0%
Injectable agents	10,292	1,221	23.512	-0.503	Dominated	0%	0%
Bladder neck needle	10,803	1,732	23.312	-0.702	Dominated	0%	0%
Open-colpo	11,605	2,535	23.839	-0.175	Dominated	6.0%	7.0%
Anterior repair	11,609	2,539	23.168	-0.847	Dominated	0%	0%
Lap-colpo	12,440	3,369	23.522	-0.492	Dominated	0%	0%
SA – estimating the incidence rate of mesh complications for Retropubic MUS (3.7%) and Transobturator MUS (2.8%) based on the data from Keltie and colleagues							
Retro-MUS	8,702		24.094			50.0%	50.0%
Trad-sling	9,087	385	24.094	-0.000	Dominated	47.0%	46.0%
Transob-MUS	10,208	1,506	23.511	-0.583	Dominated	0%	0%
Single incision sling	10,210	1,508	23.315	-0.779	Dominated	0%	0%
Injectable agents	10,278	1,576	23.550	-0.544	Dominated	0%	0%
Bladder neck needle	10,814	2,112	23.385	-0.710	Dominated	0%	0%
Anterior repair	11,605	2,903	23.211	-0.883	Dominated	0%	0%
Open-colpo	11,618	2,916	23.905	-0.189	Dominated	2.0%	3.0%
Lap-colpo	12,439	3,737	23.629	-0.465	Dominated	1.0%	1.0%
SA –assuming 10% incidence rate of mesh complications after Retropubic MUS and Transobturator MUS							
Retro-MUS	8,796		23.976			47.0%	45.0%
Trad-sling	9,093	297	23.988	0.013	23,783	50.0%	50.0%
Single incision sling	10,201	1,107	23.170	-0.818	Dominated	0%	0%
Injectable agents	10,293	1,200	23.490	-0.498	Dominated	0%	0%

Javanbakht M, *et al. BMJ Open* 2020; 10:e035555. doi: 10.1136/bmjopen-2019-035555

Transob-MUS	10,295	1,202	23.370	-0.618	Dominated	0%	0%
Bladder neck needle	10,804	1,711	23.307	-0.681	Dominated	0%	0%
Anterior repair	11,606	2,513	23.122	-0.866	Dominated	0%	0%
Open-colpo	11,618	2,524	23.826	-0.162	Dominated	3.0%	5.0%
Lap-colpo	12,438	3,345	23.491	-0.497	Dominated	0%	0%
SA –assuming 20% incidence rate of mesh complications after Retropubic MUS and Transobturator MUS							
Retro-MUS	8,906		24.021			42.0%	40.0%
Trad-sling	9,098	192	24.090	0.069	2,777	50.0%	49.0%
Single incision sling	10,200	1,102	23.317	-0.773	Dominated	0%	0%
Injectable agents	10,284	1,186	23.572	-0.519	Dominated	0%	0%
Transob-MUS	10,415	1,317	23.458	-0.633	Dominated	0%	0%
Bladder neck needle	10,806	1,708	23.417	-0.673	Dominated	0%	0%
Anterior repair	11,617	2,519	23.258	-0.833	Dominated	0%	0%
Open-colpo	11,623	2,525	23.944	-0.147	Dominated	8.0%	11.0%
Lap-colpo	12,440	3,342	23.653	-0.437	Dominated	0%	0%

Table S7 Results from deterministic sensitivity analyses-incorporating longer duration for persistent pain complication (lifetime time horizon)

Strategy	Cost (£)	Incremental cost (£)	QALY	Incremental QALY	ICER (£) (ΔCost/ΔQALY)	Probability cost-effective for different WTP thresholds (%)	
						£20,000	£30,000
Base-case analysis- assuming that persistent pain complication will last on average for 6 months							
Retro-MUS	8,666		24.005			51.0%	48.0%
Trad-sling	9,071	405	24.014	0.009	45,340	43.0%	45.0%
Transob-MUS	10,174	1,103	23.435	-0.580	Dominated	0%	0%
Single incision sling	10,189	1,118	23.221	-0.793	Dominated	0%	0%
Injectable agents	10,292	1,221	23.512	-0.503	Dominated	0%	0%
Bladder neck needle	10,803	1,732	23.312	-0.702	Dominated	0%	0%
Open-colpo	11,605	2,535	23.839	-0.175	Dominated	6.0%	7.0%
Anterior repair	11,609	2,539	23.168	-0.847	Dominated	0%	0%
Lap-colpo	12,440	3,369	23.522	-0.492	Dominated	0%	0%
SA – assuming that persistent pain complication will last on average for 36 months							
Retro-MUS	8,662		23.902			39.0%	35.0%

Javanbakht M, *et al. BMJ Open* 2020; 10:e035555. doi: 10.1136/bmjopen-2019-035555

Trad-sling	9,097	435	23.911	0.009	30,466	47.0%	42.0%
Transob-MUS	10,174	1,077	23.304	-0.607	Dominated	0%	0%
Single incision sling	10,194	1,097	23.182	-0.729	Dominated	0%	0%
Injectable agents	10,299	1,202	23.533	-0.378	Dominated	0%	0%
Bladder neck needle	10,814	1,717	23.341	-0.570	Dominated	0%	0%
Open-colpo	11,593	2,496	23.842	-0.069	Dominated	12.0%	20.0%
Anterior repair	11,607	2,510	23.122	-0.789	Dominated	0%	0%
Lap-colpo	12,437	3,340	23.564	-0.347	Dominated	2.0%	3.0%
SA – assuming that persistent pain complication will last on average for 60 months							
Retro-MUS	8,677		23.781			51.0%	41.0%
Traditional sling	9,075	397	23.721	-0.060	Dominated	25.0%	24.0%
Single incision sling	10,186	1,509	23.162	-0.619	Dominated	0%	0%
Transob-MUS	10,187	1,509	23.201	-0.580	Dominated	0%	0%
Injectable agents	10,283	1,606	23.474	-0.307	Dominated	2.0%	2.0%
Bladder neck needle	10,806	2,129	23.303	-0.478	Dominated	0%	0%
Anterior repair	11,606	2,928	23.153	-0.628	Dominated	0%	0%
Open-colpo	11,614	2,937	23.843	0.062	53,948	22.0%	33.0%
Lap-colpo	12,432	818	23.574	-0.256	Dominated	0%	0%

Table S8 Results from deterministic sensitivity analyses-incorporating higher incidence rate for persistent pain complication after MUS procedures (lifetime time horizon)

**Strategy**	**Cost (£)**	**Incremental cost (£)**	**QALY**	**Incremental QALY**	**ICER (£) (ΔCost/ΔQALY)**	**Probability cost-effective for different WTP thresholds (%)**	
						**£20,000**	**£30,000**
Base-case analysis- assuming that incidence rate of persistent pain after Retropubic MUS and Transobturator MUS are 5.09% and 4.93%							
Retro-MUS	8,666		24.005			51.0%	48.0%
Trad-sling	9,071	405	24.014	0.009	45,340	43.0%	45.0%
Transob-MUS	10,174	1,103	23.435	-0.580	Dominated	0%	0%
Single incision sling	10,189	1,118	23.221	-0.793	Dominated	0%	0%
Injectable agents	10,292	1,221	23.512	-0.503	Dominated	0%	0%
Bladder neck needle suspensions	10,803	1,732	23.312	-0.702	Dominated	0%	0%
Open-colpo	11,605	2,535	23.839	-0.175	Dominated	6.0%	7.0%
Anterior repair	11,609	2,539	23.168	-0.847	Dominated	0%	0%

Javanbakht M, *et al. BMJ Open* 2020; 10:e035555. doi: 10.1136/bmjopen-2019-035555

Lap-colpo	12,440	3,369	23.522	-0.492	Dominated	0%	0%
SA – assuming that incidence rate of persistent pain after Retropubic MUS and Transobturator MUS is 10%							
Retro-MUS	8,695		24.072			40.0%	39.0%
Trad-sling	9,089	394	24.152	0.080	4,730	57.0%	56.0%
Transob-MUS	10,916	1,107	23.533	-0.619	Dominated	0%	0%
Single incision sling	10,201	1,112	23.531	-0.621	Dominated	0%	0%
Injectable agents	10,284	1,195	23.601	-0.551	Dominated	0%	0%
Bladder neck needle suspensions	10,812	1,723	23.471	-0.681	Dominated	0%	0%
Anterior repair	11,610	2,521	23.274	-0.878	Dominated	0%	0%
Open-colpo	11,620	2,531	23.972	-0.180	Dominated	3.0%	5.0%
Lap-colpo	12,455	3,366	23.673	-0.479	Dominated	0%	0%
SA – assuming that incidence rate of persistent pain after Retropubic MUS and Transobturator MUS is 20%							
Retro-MUS	8,667		23.973			27.0%	26.0%
Trad-sling	9,079	412	24.084	0.111	3,590	64.0%	63.0%
Single incision sling	10,184	1,104	23.274	-0.810	Dominated	0%	0%
Transob-MUS	10,184	1,105	23.432	-0.652	Dominated	0%	0%
Injectable agents	10,261	1,182	23.553	-0.531	Dominated	0%	0%
Bladder neck needle suspensions	10,802	1,723	23.342	-0.742	Dominated	0%	0%
Anterior repair	10,597	2,518	23.191	-0.893	Dominated	0%	0%
Open-colpo	11,600	2,521	23.871	-0.213	Dominated	9.0%	11.0%
Lap-colpo	12,420	3,341	23.562	-0.522	Dominated	0%	0%

Table S9 Results from deterministic sensitivity analyses- applying higher incidence rates and longer duration for persistent pain after MUS procedures (lifetime time horizon)

Strategy	Cost (£)	Incremental cost (£)	QALY	Incremental QALY	ICER (£) (ΔCost/ΔQALY)	Probability cost-effective for different WTP thresholds (%)	
						£20,000	£30,000
Base-case analysis- assuming that incidence rate of persistent pain after Retropubic MUS and Transobturator MUS are 5.09% and 4.93% and average duration of persistent pain is 6 months							
Retro-MUS	8,666		24.005			51.0%	48.0%
Trad-sling	9,071	405	24.014	0.009	45,340	43.0%	45.0%
Injectable agents	10,174	1,103	23.435	-0.580	Dominated	0%	0%
Single incision sling	10,189	1,118	23.221	-0.793	Dominated	0%	0%

Transob-MUS	10,292	1,221	23.512	-0.503	Dominated	0%	0%
Bladder neck needle	10,803	1,732	23.312	-0.702	Dominated	0%	0%
Open-colpo	11,605	2,535	23.839	-0.175	Dominated	6.0%	7.0%
Anterior repair	11,609	2,539	23.168	-0.847	Dominated	0%	0%
Lap-colpo	12,440	3,369	23.522	-0.492	Dominated	0%	0%
SA – assuming that incidence rate of persistent pain after Retropubic MUS and Transobturator MUS are 20% and average duration of persistent pain is 60 months							
Retro-MUS	8,688		23.131			0%	0%
Trad-sling	9,079	391	23.752	0.621	630	46.0%	45.0%
Single incision sling	10,186	1,107	23.181	-0.571	Dominated	1.0%	1.0%
Transob-MUS	10,198	1,119	22.523	-1.229	Dominated	0%	0%
Injectable agents	10,301	1,222	23.511	-0.241	Dominated	11.0%	10.0%
Bladder neck needle	10,817	1,738	23.341	-0.411	Dominated	0%	0%
Open-colpo	11,599	2,520	23.834	0.082	30,319	39.0%	39.0%
Anterior repair	11,607	8	23.153	-0.681	Dominated	0%	0%
Lap-colpo	12,432	833	23.552	-0.282	Dominated	3.0%	5.0%

Table S10 Results from deterministic sensitivity analyses-applying different values for short and long term cure rates after retropubic MUS (lifetime time horizon)

Strategy	Cost (£)	Incremental cost (£)	QALY	Incremental QALY	ICER (£) (ΔCost/ΔQALY)	Probability cost-effective for different	
						£20,000	£30,000
Base-case analysis – applying estimated mean values from the meta-analysis for short and long-term cure rates after Retropubic MUS							
Retro-MUS	8,666		24.005			51.0%	48.0%
Trad-sling	9,071	405	24.014	0.009	45,340	43.0%	45.0%
Transob-MUS	10,174	1,103	23.435	-0.580	Dominated	0%	0%
Single incision sling	10,189	1,118	23.221	-0.793	Dominated	0%	0%
Injectable agents	10,292	1,221	23.512	-0.503	Dominated	0%	0%
Bladder neck needle	10,803	1,732	23.312	-0.702	Dominated	0%	0%
Open-colpo	11,605	2,535	23.839	-0.175	Dominated	6.0%	7.0%
Anterior repair	11,609	2,539	23.168	-0.847	Dominated	0%	0%
Lap-colpo	12,440	3,369	23.522	-0.492	Dominated	0%	0%
SA – applying values from Ward and colleagues,(1) for short and long-term cure rates after Retropubic MUS							

Javanbakht M, *et al. BMJ Open* 2020; 10:e035555. doi: 10.1136/bmjopen-2019-035555

Retro-MUS	7,848		24.123			97.0%	95.0%
Trad-sling	9,195	1,347	23.682	-0.441	Dominated	1.0%	2.0%
Transob-MUS	10,111	2,263	23.222	-0.901	Dominated	0%	0%
Single incision sling	10,176	2,328	23.161	-0.962	Dominated	0%	0%
Injectable agents	10,290	2,442	23.484	-0.639	Dominated	0%	0%
Bladder neck needle	10,831	2,983	23.304	-0.819	Dominated	0%	0%
Anterior repair	11,599	3,751	23.131	-0.992	Dominated	0%	0%
Open-colpo	11,761	3,914	23.751	-0.372	Dominated	2.0%	3.0%
Lap-colpo	12,467	4,619	23.513	-0.610	Dominated	0%	0%
SA – applying data from Song and colleagues,(2) for short and long-term cure rates after Retropubic MUS							
Retro-MUS	8,107		24.014			91.0%	87.0%
Trad-sling	9,235	1,128	23.674	-0.340	Dominated	4.0%	4.0%
Transob-MUS	10,160	2,053	23.182	-0.832	Dominated	0%	0%
Single incision sling	10,204	2,097	23.143	-0.872	Dominated	0%	0%
Injectable agents	10,288	2,181	23.481	-0.533	Dominated	0%	0%
Bladder neck needle	10,830	2,722	23.291	-0.723	Dominated	0%	0%
Anterior repair	10,611	3,504	23.122	-0.892	Dominated	0%	0%
Open-colpo	11,794	3,686	23.731	-0.283	Dominated	4.0%	8.0%
Lap-colpo	12,494	4,387	23.513	-0.501	Dominated	1.0%	1.0%

Javanbakht M, *et al. BMJ Open* 2020; 10:e035555. doi: 10.1136/bmjopen-2019-035555

Value of Information Analysis Results

Table S11 **Results from Expected Value of Partial Perfect Information Analysis**

Parameters	Per person EVPPI per year (£)	EVPPI for UK per year (£)
Health utility values	168	2,520,000
Operation related mortality rates	60	900,000
Relative treatment effectiveness	1,395	20,925,000
Combinations of parameters associated with all the complications	3,973	59,595,000
Combinations of parameters associated with short-term and persistent pain	430	6,450,000
Combinations of parameters associated with short-term and persistent pain and mesh erosion/removal	1,873	28,095,000

Javanbakht M, *et al. BMJ Open* 2020; 10:e035555. doi: 10.1136/bmjopen-2019-035555

References

1. Ward KL, Hilton P. Tension-free vaginal tape versus colposuspension for primary urodynamic stress incontinence: 5-year follow up. BJOG : an international journal of obstetrics and gynaecology. 2008;115(2):226-33.
2. Song PH, Kwon DH, Ko YH, Jung HC. The Long-Term Outcomes of the Tension-free Vaginal Tape Procedure for Treatment of Female Stress Urinary Incontinence: Data from Minimum 13 Years of Follow-Up. LUTS: Lower Urinary Tract Symptoms. 2017;9(1):10-4.
3. Department of Health. Reference Costs 2017-182018.
4. Boyers D, Kilonzo M, Mostafa A, Abdel-Fattah M. Comparison of an adjustable anchored single-incision mini-sling, Ajust®, with a standard mid-urethral sling, TVT-OTM: a health economic evaluation. BJU Int. 2013;112(8):1169-77.
5. Cody J. Systematic review of the clinical effectiveness and cost-effectiveness of tension-free vaginal tape for treatment of urinary stress incontinence. 2003.
6. Urinary incontinence in women: management Clinical guideline [CG171] 2015 [Available from: https://www.nice.org.uk/guidance/cg171.
7. Curtis L, Burns A. Unit Costs of Health & Social Care 2016: The University of Kent; 2016.
8. British National Formulary. BNF Online [Available from: https://www.medicinescomplete.com/mc/?utm_source=bnforg&utm_medium=homepage&utm_campaign=medicinescomplete.
9. Manca A, Sculpher MJ, Ward K, Hilton P. A cost-utility analysis of tension-free vaginal tape versus colposuspension for primary urodynamic stress incontinence. BJOG : an international journal of obstetrics and gynaecology. 2003;110(3):255-62.
10. Haywood KL, Garratt AM, Lall R, Smith JF, Lamb SE. EuroQol EQ-5D and condition-specific measures of health outcome in women with urinary incontinence: reliability, validity and responsiveness. Qual Life Res. 2008;17(3):475-83.
11. Shepherd JP, Lowder JL, Jones KA, Smith KJ. Retropubic and transobturator midurethral slings: a decision analysis to compare outcomes including efficacy and complications. Int Urogynecol J Pelvic Floor Dysfunct. 2010;21(7):787-93.

Javanbakht M, *et al. BMJ Open* 2020; 10:e035555. doi: 10.1136/bmjopen-2019-035555
