# Supplementary material for: Economic evaluation of surgical treatments for women with stress urinary incontinence: a cost-utility and value of information analysis
Source: BMJ Open. 2020 Jun 11;10(6):e035555. doi: 10.1136/bmjopen-2019-035555 (PMC7295417; doi:10.1136/bmjopen-2019-035555)
Supplement: Author's manuscript [file bmjopen-2019-035555.draft_revisions.pdf]

BMJ Open

BMJ Open is committed to open peer review. As part of this commitment we make the peer review history of every article we publish publicly available.

When an article is published we post the peer reviewers' comments and the authors' responses online. We also post the versions of the paper that were used during peer review. These are the versions that the peer review comments apply to.

The versions of the paper that follow are the versions that were submitted during the peer review process. They are not the versions of record or the final published versions. They should not be cited or distributed as the published version of this manuscript.

BMJ Open is an open access journal and the full, final, typeset and author-corrected version of record of the manuscript is available on our site with no access controls, subscription charges or pay-per-view fees (http://bmjopen.bmj.com).

If you have any questions on BMJ Open's open peer review process please email
info.bmjopen@bmj.com

Economic Evaluation of Surgical Treatments for Women with Stress Urinary Incontinence: A Cost-Utility and Value of Information Analysis

Journal:	*BMJ Open*
Manuscript ID	bmjopen-2019-035555
Article Type:	Original research
Date Submitted by the Author:	07-Nov-2019
Complete List of Authors:	Javanbakht, Mehdi; Institute of Health & Society, Newcastle University, Health Economics ; Institute of Health & Society, Newcastle University<br>Moloney, Eoin; Institute of Health & Society, Newcastle University, Health Economics Group, Institute of Health and Society<br>Brazzelli, Miriam; University of Aberdeen, Health Services Research Unit<br>Wallace, Sheila<br>Ternent, Laura; Newcastle University, Institute of Health & Society<br>Omar, Muhammad; University of Aberdeen, Academic Urology Unit/ Cochrane Incontinence Group<br>Monga, Ash ; University of Southampton<br>Saraswat, L; University of Aberdeen,<br>Mackie, Phil<br>Becker, Frauke; University of Oxford, Health Economics Research Centre<br>Imamura, Mari; University of Aberdeen<br>Hudson, Jemma; The Centre for Healthcare Randomised Trials (CHaRT), Health Services Research Unit, University of Aberdeen, Foresterhill<br>Shimonovich, Michal ; University of Aberdeen<br>Maclennan, Graeme; University of Aberdeen, Health Services Research Unit<br>Vale, Luke; Newcastle University, Health Economics Group, Institute of Health and Society<br>Craig, Dawn; Institute of Health & Society, Newcastle University, Health Economics Group, Institute of Health and Society
Keywords:	Health economics < HEALTH SERVICES ADMINISTRATION & MANAGEMENT, Urinary incontinences < UROLOGY, Adult surgery < SURGERY

SCHOLARONE™
Manuscripts

I, the Submitting Author has the right to grant and does grant on behalf of all authors of the Work (as defined in the below author licence), an exclusive licence and/or a non-exclusive licence for contributions from authors who are: i) UK Crown employees; ii) where BMJ has agreed a CC-BY licence shall apply, and/or iii) in accordance with the terms applicable for US Federal Government officers or employees acting as part of their official duties; on a worldwide, perpetual, irrevocable, royalty-free basis to BMJ Publishing Group Ltd ("BMJ") its licensees and where the relevant Journal is co-owned by BMJ to the co-owners of the Journal, to publish the Work in this journal and any other BMJ products and to exploit all rights, as set out in our licence.

The Submitting Author accepts and understands that any supply made under these terms is made by BMJ to the Submitting Author unless you are acting as an employee on behalf of your employer or a postgraduate student of an affiliated institution which is paying any applicable article publishing charge ("APC") for Open Access articles. Where the Submitting Author wishes to make the Work available on an Open Access basis (and intends to pay the relevant APC), the terms of reuse of such Open Access shall be governed by a Creative Commons licence – details of these licences and which Creative Commons licence will apply to this Work are set out in our licence referred to above.

Other than as permitted in any relevant BMJ Author's Self Archiving Policies, I confirm this Work has not been accepted for publication elsewhere, is not being considered for publication elsewhere and does not duplicate material already published. I confirm all authors consent to publication of this Work and authorise the granting of this licence.

For peer review only

Economic Evaluation of Surgical Treatments for Women with Stress Urinary Incontinence: A Cost-Utility and Value of Information Analysis

Running title: Cost-Utility of Surgical Treatments for Stress Urinary Incontinence

Mehdi Javanbakht[1], Eoin Moloney[1*], Miriam Brazzelli[2], Sheila Wallace[1], Laura Ternent[1], Muhammad Imran Omar[3], Ash Monga[4], Lucky Saraswat[5], Phil Mackie[6], Frauke Becker[1], Mari Imamura[2], Jemma Hudson[2], Michal Shimonovich[2], Graeme MacLennan[2], Luke Vale[1], Dawn Craig[1]

1- Institute of Health & Society, Newcastle University, Newcastle upon Tyne, UK
2- Health Services Research Unit, University of Aberdeen, Aberdeen, UK
3- Guidelines Office Methodology Supervisor, European Association of Urology, Arnhem, Netherlands
4- University Hospitals Southampton Foundation Trust, Southampton, UK
5- Aberdeen Royal Infirmary, Aberdeen, UK
6- Scottish Public Health Network, Head of Knowledge and Research Services, NHS Health Scotland

***Corresponding author:** Eoin Moloney
Institute of Health & Society, Baddiley-Clark Building, Newcastle University, Richardson Road, Newcastle upon Tyne, NE2 4AX; E-mail: eoin.moloney@newcastle.ac.uk; Telephone: +44 (0) 191 208 7359

Word count: abstract: 296; Manuscript: Approx. 3,000

For peer review only - http://bmjopen.bmj.com/site/about/guidelines.xhtml

Abstract

Objectives: Stress urinary incontinence (SUI) and stress-predominant mixed urinary incontinence (MUI) are common conditions that can have a negative impact on the quality of life of patients and serious cost implications for health care providers. The objective of this study was to assess the cost-effectiveness of nine different surgical interventions for treatment of SUI and stress-predominant MUI from an NHS and personal social services perspective in the UK.

Methods: A Markov microsimulation model was developed to compare the costs and effectiveness of nine surgical interventions. The model was informed by undertaking a systematic review of clinical effectiveness and network meta-analysis. The main clinical parameters in the model were the cure and incidence rates of complications after different interventions. The outcomes from the model were expressed in terms of cost per quality-adjusted life-years (QALYs) gained. In addition, expected value of perfect information (EVPI) analyses were conducted to quantify the main uncertainties facing decision-makers.

Results: The base-case results suggest that Retropubic mid-urethral sling (Retro-MUS) is the most cost-effective surgical intervention over a 10-year and lifetime time horizon. The probabilistic results show that Retro-MUS and traditional sling are the interventions with the highest probability of being cost-effective across all willingness to pay thresholds over a lifetime time horizon. The value of information analysis results suggest that the largest value appears to be in removing uncertainty around the incidence rates of complications, the relative treatment effectiveness, and health utility values.

Conclusions: Although Retro-MUS appears, at this stage, to be a cost-effective intervention, research is needed on possible long-term complications of all surgical treatments to provide reassurance of safety, or earlier warning of unanticipated adverse effects. The value of information analysis supports the need, as a first step, for further research to improve our knowledge of the actual incidence of complications.

Key Words: Stress urinary incontinence, Surgery, Economic evaluation, Cost-effectiveness analysis, Markov microsimulation.

For peer review only - http://bmjopen.bmj.com/site/about/guidelines.xhtml

Article Summary

Strengths and limitations of this study

- An economic model has been developed to consider the relative cost-effectiveness of nine different surgical interventions for stress urinary incontinence and stress-predominant mixed urinary incontinence.
- The model has been informed and populated based on a comprehensive systematic review and network meta-analysis of the clinical effectiveness of surgical interventions.
- Uncertainty in the model results has been explored through a probabilistic sensitivity analysis, and value of information analysis to consider the value of future research.
- Due to limited long-term data on effectiveness, short-term data were extrapolated over a longer-term time horizon.

For peer review only

For peer review only - http://bmjopen.bmj.com/site/about/guidelines.xhtml

Introduction

Stress urinary incontinence (SUI) in women is a disabling and common condition which reduces quality of life and causes significant stress and concern amongst individual patients (1, 2). Commonly defined as the 'involuntary leakage of urine' due to activities such as coughing, sneezing or exercising, the prevalence of SUI varies (20 to 50%) but is greater in women who have had children and in older women (3, 4). Associated conditions include urge urinary incontinence (UUI), which is characterised by a sudden and uncontrollable need to urinate (i.e. urgency), and mixed urinary incontinence (MUI) which refers to a combination of the symptoms associated with SUI and UUI (2). Where the symptoms of SUI dominate those of UUI in a patient with MUI, stress-predominant MUI is said to exist.

In addition to the emotional impact that SUI can have on patients, there are serious cost implications associated with the condition, both for the health care provider and the individual patient (5). Amongst the previous studies to have investigated the economic burden of the condition, one published study based on data from three European countries suggested that the total cost of SUI is approximately £818 million in the UK (2), whilst another suggested a health care cost to the UK NHS of £117 million per year (SUI only) (5). In addition to the health service costs, patients may incur out-of-pocket expenses due to the need to purchase consumables and services not available through their health care provider (5).

Conservative treatment with physiotherapy to deliver pelvic floor muscle exercises and bladder training is the first line of treatment for patients with SUI. However, when this fails, or when insufficient relief from symptoms is achieved, surgery is the recommended treatment (6). The aim of surgery is to support or partially obstruct the bladder neck and/or urethra, thus preventing the leakage of urine on exertion (7). Surgical techniques have evolved over time, moving most recently to more minimally invasive techniques, such as mid-urethral slings (MUS) where a synthetic mesh or tape is placed under the urethra and secured using a number of different methods (8). However, it is unclear if these newly available treatments such as retropubic mid-urethral sling (Retro-MUS), single incision sling and injectable bulking agents really result in equivalent or better cost and health outcomes than older operations that were previously available (such as anterior vaginal repair or colposuspension).

The wide range of surgical operations available, the different techniques used to perform these operations, and the lack of a consensus amongst surgeons regarding which approach to use, make it challenging to establish which procedure should be used to treat SUI or stress-predominant mixed urinary incontinence MUI. Health economic techniques have become increasingly important as a tool to help inform decisions between treatments and interventions. They allow for the consideration of all relevant costs (from the specified perspective) and clinical outcomes associated with the treatments being compared. Findings can then be used to make a decision on how best to allocate resources in the most cost-effective way. This study was conducted as part of a larger project to evaluate the cost-effectiveness of Retro-MUS versus eight different comparator surgical interventions for the treatment of women with SUI or stress-predominant MUI (9).

Methods

Study Design

A Markov microsimulation (MM) model was developed to assess cost-effectiveness. The model was informed by a review of published economic evaluations, and the findings of a systematic review of clinical effectiveness and network meta-analyses (including 120 trials) conducted as part of the larger project to estimate the relative effectiveness of nine different surgical treatments (9). Retro-MUS was chosen as the intervention against which all other surgical interventions would be compared, due to the fact that it is the most common type of surgery and it is widely regarded as standard practice (10). The eight comparators were: 1) Anterior vaginal repair or anterior colporrhaphy (Anterior repair), 2) Bladder neck needle suspensions (Bladder neck needle), 3) Open abdominal retropubic colposuspension (Open-colpo), 4) Laparoscopic retropubic colposuspension (Lap-colpo), 5) Traditional sub-urethral retropubic

sling procedures (Trad-sling), 6) Transobturator mid-urethral sling (Transob-MUS), 7) Single incision sling procedures (Single incision sling) and 8) Peri-urethral injections bulking agents (Injectable agents).

Health outcomes from the model were expressed in terms of quality-adjusted life-years (QALYs) and costs in 2015/16 GBP (£). The costs were estimated from the NHS and personal social service perspective. Both costs and QALYs were evaluated over 1-year, 10-year and lifetime time horizons and discounted using a 3.5% annual discount rate (11). The expected cost and QALYs for each of the strategies were compared using incremental cost-effectiveness ratios (ICERs) where appropriate.

Target Population

The model was based on a hypothetical group of women (45-55 years) with SUI or stress-predominant MUI, undergoing surgical intervention for their condition.

Model Structure

The model structure is presented in Figure 1. Treatment history for both SUI/stress-predominant MUI and UUI was recorded for each modeled woman and used to define the patient transitions to different health states and treatment types. The model assumes that patients can receive a maximum of three surgical treatments for treatment of SUI/stress-predominant MUI, which includes the initial surgery plus two subsequent re-treatments. If all three surgeries fail, patients have to manage their symptoms using containment products. The model allows for individuals to elect to use containment products at any point after the initial treatment has failed. Patients with stress-predominant MUI who still have UUI after SUI is cured, or those who develop UUI due to a surgery, i.e. as a complication following the procedure, will receive three lines of treatment based on clinical guidelines including first-line (Bladder training), second-line (Medication) and third-line treatment (Botulinum toxin A). The model was developed in TreeAge Pro® package (TreeAge Software, Inc., Williamstown, MA, USA) (12).

Figure 1 Markov model structure

Model Inputs

Effectiveness of surgical treatments

The relative effectiveness of surgical treatments, in terms of subjective cure rates, were based on the results of a network meta-analysis (9). Table 1 describes the mean and median odds ratios (ORs) for different surgical treatments vs Retro-MUS. The absolute cure rates were calculated in the model by combining the information on relative cure rates described in Table 1 with the absolute cure rates for Retro-MUS, presented in Table 2. The long-term recurrence rates after Retro-MUS were estimated using parametric survival models. Scale and shape parameters were estimated for a Weibull hazard function using the reported cure rates at 1 and 5 years (online supplementary files 1, 2, 3, 4, respectively). In the base-case analysis it was assumed that 75% of women whose first treatment was not successful would seek re-treatment and 30% of women whose first retreatment failed would seek a second retreatment (13).

Table 1 Odds ratios of cure rates for surgical interventions compared with Retro-MUS

Treatment	Mean	Median	Lower bound of 95% Crl	Upper bound of 95% Crl
Transob-MUS vs Retro-MUS	0.742	0.738	0.588	0.923
Open-colpo vs Retro-MUS	0.874	0.853	0.544	1.325

For peer review only - http://bmjopen.bmj.com/site/about/guidelines.xhtml

Lap-colpo vs Retro-MUS	0.605	0.58	0.315	1.046
Trad-sling vs Retro-MUS	1.106	1.061	0.623	1.846
Single incision vs Retro-MUS	0.511	0.504	0.36	0.699
Bladder neck needle vs Retro-MUS	0.368	0.34	0.154	0.745
Anterior repair vs Retro-MUS	0.235	0.22	0.105	0.452

Anterior repair: Anterior vaginal repair (anterior colporrhaphy); Bladder neck needle: Bladder neck needle suspensions; CrI: Credible interval; Lap-colpo: Laparoscopic retropubic colposuspension; Open-colpo: Open abdominal retropubic colposuspension; Trad-sling: Traditional suburethral retropubic sling procedures; MUS: Mid-urethral sling; Retro-MUS: Retropubic mid-urethral sling; Transob-MUS: Transobturator mid-urethral sling; Single incision: Single incision sling procedures ('mini-slings')

Table 2 Estimation of absolute cure rates after Retro-MUS in different time points-results from meta-analysis

Time	Median	95% Crl	Number of studies	Number of participants
6 months	0.776	(0.175, 0.983)	17	908
12 months	0.841	(0.214, 0.990)	44	2882
24 months	0.784	(0.454, 0.941)	6	315
36 months	0.341	(0.001, 0.995)	5	205
60 months	0.329	(0.005, 0.979)	3	377

Complication and mortality rates after surgical treatments

The model incorporated only severe complications/adverse events that are most important in terms of their effect on patient's quality of life, cost and duration of impact. To estimate complication incidence rates, random-effects meta-analysis models were fitted to data from the RCTs identified within the systematic review (14). All of the complications included in the model, and estimated incidence rates, are presented in online supplementary file 5. Age-specific all-cause mortality rates were derived from general population mortality statistics reported in national life tables (15). The effectiveness of the three lines of treatments for UUI were informed by the results from previous meta-analyses (6, 16).

Resource use and unit costs

Surgeries for SUI vary in terms of the complexity of the procedure and the setting in which surgery is conducted. For patients undergoing Anterior repair, Bladder neck needle, Open-colpo, Lap-colpo, and Trad-sling, surgery would typically be conducted in an inpatient setting. Patients undergoing Retro-MUS, Transob-MUS, Single incision sling and Injectable agents would typically be treated in a day-case setting. In addition to the cost of individual surgeries, costs associated with complementary tests, treatments and consultations that would typically be carried out in advance of, and following, each surgery were also considered. Unit costs were derived from UK NHS reference costs (17), Personal Social Services Research Unit (PSSRU) (18) and British National Formulary (BNF) (19) for medication. The price year of the analysis was 2015/16. All costs are reported in online supplementary file 6.

Health Utility

The baseline health utility value for pre-treatment SUI was derived from a previous UK-based economic evaluation comparing two types of surgical intervention for this condition (20). The utility value for a successful treatment was derived from a UK-based study exploring health outcomes in women with

For peer review only - http://bmjopen.bmj.com/site/about/guidelines.xhtml

urinary incontinence (21). Utility decrements associated with each complication included in the model were obtained from previous studies (22, 23) (online supplementary file 7).

Patient and Public Involvement

As part of the larger project (9), patient and public involvement (PPI) was included to understand patient's thoughts and feelings regarding their condition and treatment and to help add context to the review and meta-analysis of clinical effectiveness. However, no additional PPI was sought for this modelling work.

Analysis and Sensitivity analyses

Both deterministic and probabilistic sensitivity analyses (PSA) were used to explore parameter and other forms of uncertainty surrounding the estimates of cost-effectiveness. Deterministic sensitivity analyses including; applying higher incidence rates of mesh complications, longer durations of persistent pain complication, higher incidence rates for persistent pain complication post MUS procedures, and applying different values for short and long-term cure rates after retropubic MUS were performed to determine the impact of changing key parameters and assumptions on the model results. The PSA was run with 10,000 simulations for each patient, and cost-effectiveness acceptability curves (CEACs) were produced in order to identify the probability of the different surgeries being cost-effective across a range of willingness-to-pay (WTP) thresholds. The economic model was used to quantify the main uncertainties facing decision-makers and to help inform decisions about the direction of future research. This was explored through value of information (VOI) analysis methods: expected value of perfect information (EVPI) and expected value of partial perfect information (EVPPI) analysis. In total, it was assumed that in the UK 15,000 surgical treatments are conducted annually for the treatment of SUI.

Results

Base-case analysis

The base-case analysis results are presented in Table 3, and in the form of a CEAC in Figure 2. The table reports strategies from the least to the most costly. Over a lifetime time horizon, Retro-MUS is, on average, the least costly (£8,099) and the second most effective (24.22 QALYs) surgical treatment. All other surgical treatments are dominated as they are more costly and less effective than Retro-MUS, except for Trad-sling which is marginally more effective (+ 0.01 QALYs). Over a 10-year time horizon, Retro-MUS remains the dominant strategy. However, over a 1-year time horizon single incision sling is dominant compared to all other strategies, except for Open-colpo which is marginally more effective (+ 0.01 QALYs).

Figure 2 shows that over a lifetime time horizon, Retro-MUS has a greater than 26% probability of being cost-effective at a WTP threshold value of £20,000. Given the number of comparators, if the interventions were comparable we would expect an 11% chance of each being cost-effective. The only other strategies with reasonably sized probabilities of being cost-effective are Trad-sling (approximately 27% across all WTP values presented, suggesting not much difference between Retro-MUS and Trad-sling) and Open-colpo (approximately 14-16% across all WTP thresholds above £10,000). All other strategies have a less than 10.5% probability of being cost-effective across all WTP values presented.

Table 3 Results of the probabilistic analysis 1-, 10 years and lifetime time horizons

Time horizon	Strategy	Cost (£)	Incremental cost (£)	QALY	Incremental QALY	ICER (£) (ΔCost/ΔQALY)	Probability of being cost-effective at

For peer review only

For peer review only - http://bmjopen.bmj.com/site/about/guidelines.xhtml

							different threshold (%)	
							£20,000	£30,000
1 Year	Single incision sling	1,953		0.76			96.6%	92.3%
	Retro-MUS	2,310	357	0.75	-0.01	Dominated	2.9%	5.6%
	Transob-MUS	2,352	399	0.75	-0.01	Dominated	0.5%	1.4%
	Bladder neck needle suspensions	2,756	803	0.75	-0.01	Dominated	0.1%	0.5%
	Trad-sling	2,772	819	0.72	-0.04	Dominated	0.0%	0.0%
	Injectable agents	2,848	895	0.74	-0.02	Dominated	0.0%	0.1%
	Anterior vaginal repair	3,249	1,296	0.76	0.00	Dominated	0.0%	0.2%
	Open-colpo	4,710	2,757	0.77	0.01	233,209	0.0%	0.0%
	Lap-colpo	4,804	95	0.76	-0.01	Dominated	0.0%	0.0%
10 Years	Retro-MUS	4,649		7.33			51.0%	44.9%
	Trad-sling	5,235	585	7.28	-0.05	Dominated	20.4%	20.5%
	Single incision sling	5,274	625	7.14	-0.19	Dominated	3.7%	3.5%
	Transob-MUS	5,414	765	7.2	-0.13	Dominated	4.3%	4.4%
	Injectable agents	5,676	1,027	7.19	-0.14	Dominated	8.8%	9.5%
	Bladder neck needle suspensions	5,958	1,309	7.14	-0.19	Dominated	4.0%	4.3%
	Anterior vaginal repair	6,655	2,006	7.11	-0.22	Dominated	1.8%	2.4%
	Open-colpo	7,375	2,725	7.29	-0.04	Dominated	4.7%	8.1%
	Lap-colpo	7,818	3,169	7.2	-0.13	Dominated	1.3%	2.5%
Life time	Retro-MUS	8,099		24.22			25.8%	24.6%
	Trad-sling	8,522	423	24.22	0.01	60,863	27.0%	26.2%
	Injectable agents	9,554	1,032	23.86	-0.36	Dominated	10.5%	10.5%
	Single incision sling	9,649	1,127	23.59	-0.63	Dominated	3.2%	3.2%
	Transob-MUS	9,665	1,142	23.71	-0.51	Dominated	4.1%	4.1%
	Bladder neck needle suspensions	10,125	1,603	23.69	-0.53	Dominated	5.4%	5.4%
	Open-colpo	10,977	2,455	24.10	-0.12	Dominated	14.1%	15.0%
	Anterior vaginal repair	11,057	2,535	23.54	-0.69	Dominated	3.9%	4.1%
	Lap-colpo	11,797	3,274	23.83	-0.40	Dominated	6.2%	6.8%

Anterior repair: Anterior vaginal repair (anterior colporrhaphy); Bladder neck needle: Bladder neck needle suspensions; CrI: Credible interval; Lap-colpo: Laparoscopic retropubic colposuspension; Open-colpo: Open abdominal retropubic colposuspension; Trad-sling: Traditional suburethral retropubic sling procedures; MUS: Mid-urethral sling; Retro-MUS: Retropubic mid-urethral sling; Transob-MUS: Transobturator mid-urethral sling; Single incision: Single incision sling procedures ('mini-slings')

Figure 2 Cost-effectiveness acceptability curves for the nine surgical treatments: lifetime time horizon

Sensitivity analysis (SA) and VOI analysis

In the first SA, results from the study by Keltie et al. (24) were used to inform the mesh complications incidence rates after Retro-MUS and Transob-MUS. Results from this SA show that Retro-MUS

For peer review only - http://bmjopen.bmj.com/site/about/guidelines.xhtml

For peer review only

remains the most cost-effective option. Results from the second SA show that when a longer duration for persistent pain is assumed (3 and 5 years), the probability of Retro-MUS being the most cost-effective option decreases and the respective probability for Open-colpo increases. Furthermore, results from the third SA show that when a higher incidence rate (10% and 20%) of persistent pain after Retro-MUS and Transob-MUS is assumed, the probability of Retro-MUS being the most cost-effective option decreases and the respective probability for Trad-sling increases. Detailed results from all sensitivity analyses are reported in online supplementary files 8, 9, 10, 11, 12, respectively.

The VOI analyses results show that the EVPI per patient is £11,180 and the estimated EVPI for the UK population for 1 year is £167.7 million. This figure increases as the time horizon (or period of time over which the information would be useful) is increased (Table 4). Results from the EVPPI analysis also show that the largest value appears to be in removing uncertainty around the incidence rate of complications, treatment effectiveness, and health utility parameters.

Table 4 Results from value of information analysis

**The expected value of removing all current decision uncertainty**	**Overall EVPI (£)**	**Overall EVPI (QALY)**
Per person affected by the decision	11,180	0.56
Per year in UK assuming 15000 persons affected per year	167,700,000	8,385
Over 5 years	838,500,000	41,930
Over 10 years	1,677,000,000	83,850
Over 15 years	2,516,000,000	125,800
Over 20 years	3,354,000,000	167,700

Discussion

To our knowledge, this economic evaluation is the most comprehensive assessment of the cost-effectiveness of surgical interventions for the treatment of SUI or stress-predominant MUI in women. Due to limited long-term clinical data, the results are uncertain but suggest that Retro-MUS is the least costly and most cost-effective surgical intervention over 10-year and lifetime time horizons; results which are largely driven by the lower initial cost of Retro-MUS. This is primarily due to the fact that this procedure is conducted in a day-case setting, and there is a lower chance of needing repeat surgery due to its higher cure rate compared to all other surgical treatments (except for Trad-sling).

One of the strengths of our economic model is the evaluation of *nine* different surgical treatments in one study, informed by data from a comprehensive evidence synthesis and network meta-analysis where all the direct and indirect evidence (120 trials) were used to estimate the relative effectiveness of different surgical treatments in terms of cure rates (9). Very few cost-effectiveness studies in this clinical area have included complications within their analysis, despite the fact that the incidence of each complication can have an impact on the woman's quality of life, and result in costs incurred for the health system. Therefore, in the present study the impact that complications such as infection, urge urinary incontinence, voiding difficulties, bladder or urethral perforation, mesh removal, short-term pain and persistent pain have on costs and effects have been incorporated into the model and explored in sensitivity analysis. Approximately 50% of women who have SUI (4) also suffer from UUI symptoms; given that UUI potentially affects the woman's quality of life more than SUI (25), treatment pathways associated with UUI are also incorporated in the model to more accurately estimate QALYs for this patient group. The analysis was conducted in accordance with the ISPOR modelling guidelines (26) and extensive sensitivity analyses were undertaken to explore and characterise uncertainty in the

For peer review only - http://bmjopen.bmj.com/site/about/guidelines.xhtml

model parameters. Our results were generally robust to the sensitivity analyses performed and are comparable to the findings of other published studies.

There are a number of published cost-effectiveness analyses evaluating some of the surgeries that we have assessed, the general findings of which are presented to allow for comparison. Two studies compared the single incision versus mid-urethral sling procedures over a 1-year time horizon, concluding that single incision was less costly and of similar effectiveness (27, 28). A UK study compared the cost-effectiveness of Retro-MUS (Tension-free Vaginal Tape (TVT)) versus open colposuspension, laparoscopic colposuspension, Trad-sling and injectable agents, concluding that TVT dominated Open-colpo over a 5-year time horizon (29). A cost–utility analysis in the UK to assess the cost-effectiveness of TVT compared with open Burch colposuspension found that TVT was less costly and more effective (20). A further study to assess the cost-effectiveness of TVT versus laparoscopic mesh colposuspension concluded that TVT was more cost-effective over a 1-year time horizon (30). Although there are limitations with many of these studies and heterogeneity present in the methods, the results from all of the above studies are largely in agreement with the findings from our economic model; generally supporting the conclusion that Retro-MUS (TVT) is likely to be the most cost-effective option when compared to the other types of surgeries for the treatment of SUI and stress-predominant MUI.

Results from the VOI analysis indicate that further research should focus on adverse events that, however rare, can have a detrimental impact on women's quality of life when they do occur (e.g. tape extrusion/exposure).

Limitations

One of the main limitations of the current study is the lack of long-term data which necessitated the extrapolation of relatively short-term data to 10 years, and over the lifetime, of women included in the analysis. Therefore, the results presented would only apply in a situation where relative differences in the effectiveness of Retro-MUS compared with the comparators do not change with longer follow-up. The long-term incidence of complications after the surgical treatments are also currently unknown. All of the estimated incidence rates for complications after each surgical treatment were based on data from trials with relatively short-term follow-up times. We tested the impact of possible higher incidence rates of some of the complications on the results, to incorporate and explore some of this uncertainty within our model. Finally, the economic analysis included costs to the NHS only. As highlighted earlier, individual patients also buy different products and incur the costs of containment products themselves; a future analysis may also need to consider these out-of-pocket costs to patients.

Conclusions

The results of this study suggest that, in the medium-and long-term, Retro-MUS is less costly and more effective than all other surgical interventions for the treatment of SUI; therefore, it is a dominant strategy. However, the results should be interpreted with caution as the long-term performance of all surgical treatments in terms of both continence and unanticipated adverse effects is not reliably known. Therefore, the results presented are based on the extrapolation of short- and medium-term evidence.

Acknowledgements

We would like to acknowledge all those involved in the wider study exploring the effectiveness and cost-effectiveness of surgical treatments for women with stress urinary incontinence (9).

For peer review only

References

1. Kwon BE, Kim GY, Son YJ, Roh YS, You MA. Quality of life of women with urinary incontinence: a systematic literature review. International neurourology journal. 2010;14(3):133-8.
2. Papanicolaou S, Pons ME, Hampel C, Monz B, Quail D, Schulenburg MG, et al. Medical resource utilisation and cost of care for women seeking treatment for urinary incontinence in an outpatient setting. Examples from three countries participating in the PURE study. Maturitas. 2005;52 Suppl 2:S35-47.
3. Hannestad YS, Rortveit G, Sandvik H, Hunskaar S. A community-based epidemiological survey of female urinary incontinence: the Norwegian EPINCONT study. Epidemiology of Incontinence in the County of Nord-Trondelag. J Clin Epidemiol. 2000;53(11):1150-7.
4. Hunskaar S, Lose G, Sykes D, Voss S. The prevalence of urinary incontinence in women in four European countries. BJU Int. 2004;93(3):324-30.
5. Turner DA, Shaw C, McGrother CW, Dallosso HM, Cooper NJ. The cost of clinically significant urinary storage symptoms for community dwelling adults in the UK. BJU Int. 2004;93(9):1246-52.
6. Urinary incontinence in women: the management of urinary incontinence in women CG171: National Institute for Health and Care Excellence 2013 [Available from: https://www.nice.org.uk/guidance/cg171/evidence/urinary-incontinence-in-women-full-guideline-191581165.
7. Sung VW, Schleinitz MD, Rardin CR, Ward RM, Myers DL. Comparison of Retropubic versus Transobturator Approach to Midurethral Slings: A Systematic Review and Meta-Analysis. Am J Obstet Gynecol. 2007;197(1):3-11.
8. Chuang K-W, Firoozi F. Surgical Management of Stress Urinary Incontinence. In: Firoozi F, editor. Female Pelvic Surgery. New York, NY: Springer New York; 2015. p. 39-54.
9. Brazzelli M, Javanbakht M, Imamura M, Hudson J, Moloney E, Becker F, et al. The Effectiveness and cost-effectiveness of Surgical Treatments for womEn with stRess urinary incontinence: An evidence synthesis, economic evaluation and discrete choice experiment (ESTER). 2017.
10. Morling J, Wood R. Using routinely available health data to examine the provision of, and outcomes following, surgery for SUI and POP in Scotland: Technical Report Information Services Division, NHS National Services Scotland; 2015 [Available from: http://www.gov.scot/Publications/2015/10/8485/downloads.
11. Excellence NIfHaC. Guide to the methods of technology appraisal 2013 2013 [Available from: https://www.nice.org.uk/process/pmg9/resources/guide-to-the-methods-of-technology-appraisal-2013-pdf-2007975843781.
12. TreeAge Pro, TreeAge Software, Williamstown, MA 2017 [Available from: https://www.treeage.com.
13. Cody J, Wyness L, Wallace S, Glazener C, Kilonzo M, Stearns S, et al. Systematic review of the clinical effectiveness and cost-effectiveness of tension-free vaginal tape for treatment of urinary stress incontinence. Health Technol Assess. 2003;7(21):iii, 1-189.
14. Lunn DJ, Thomas A, Best N, Spiegelhalter D. WinBUGS-a Bayesian modelling framework: concepts, structure, and extensibility. Statistics and computing. 2000;10(4):325-37.
15. Zilberman D, Kaplan S, Wesseler J. The loss from underutilizing GM technologies. AgBioForum. 2015;18(3):312-9.
16. Imamura M, Abrams P, Bain C, Buckley B, Cardozo L, Cody J, et al. Systematic review and economic modelling of the effectiveness and cost-effectiveness of non-surgical treatments for women with stress urinary incontinence. Health Technol Assess. 2010;14(40):1-188, iii-iv.
17. Health Do. Reference Costs 2015-16 2016 [Available from: https://improvement.nhs.uk/resources/reference-costs/.
18. Curtis L, Burns A. Unit Costs of Health & Social Care 2016: The University of Kent; 2016.

For peer review only - http://bmjopen.bmj.com/site/about/guidelines.xhtml

19. British National Formulary. BNF Online [Available from: https://www.medicinescomplete.com/mc/?utm_source=bnforg&utm_medium=homepage&utm_campaign=medicinescomplete.
20. Manca A, Sculpher MJ, Ward K, Hilton P. A cost-utility analysis of tension-free vaginal tape versus colposuspension for primary urodynamic stress incontinence. BJOG : an international journal of obstetrics and gynaecology. 2003;110(3):255-62.
21. Haywood KL, Garratt AM, Lall R, Smith JF, Lamb SE. EuroQol EQ-5D and condition-specific measures of health outcome in women with urinary incontinence: reliability, validity and responsiveness. Qual Life Res. 2008;17(3):475-83.
22. Kunkle CM, Hallock JL, Hu X, Blomquist J, Thung SF, Werner EF. Cost utility analysis of urethral bulking agents versus midurethral sling in stress urinary incontinence. Female pelvic med. 2015;21(3):154-9.
23. Shepherd JP, Lowder JL, Jones KA, Smith KJ. Retropubic and transobturator midurethral slings: a decision analysis to compare outcomes including efficacy and complications. Int Urogynecol J Pelvic Floor Dysfunct. 2010;21(7):787-93.
24. Keltie K, Elneil S, Monga A, Patrick H, Powell J, Campbell B, et al. Complications following vaginal mesh procedures for stress urinary incontinence: an 8 year study of 92,246 women. Scientific Reports. 2017;7(1):12015.
25. Tincello D, Sculpher M, Tunn R, Quail D, van der Vaart H, Falconer C, et al. Patient Characteristics Impacting Health State Index Scores, Measured by the EQ-5D of Females with Stress Urinary Incontinence Symptoms. Value Health. 2010;13(1):112-8.
26. Caro JJ, Briggs AH, Siebert U, Kuntz KM. Modeling good research practices--overview: a report of the ISPOR-SMDM Modeling Good Research Practices Task Force--1. Value Health. 2012;15(6):796-803.
27. Costantini E, Lazzeri M, Bini V, Zucchi A, Scarponi E, Porena M. Managing female urinary incontinence: a regional prospective analysis of cost-utility ratios (curs) and effectiveness. Archivio italiano di urologia, andrologia : organo ufficiale [di] Societa italiana di ecografia urologica e nefrologica. 2014;86(2):112-7.
28. Boyers D, Kilonzo M, Mostafa A, Abdel-Fattah M. Comparison of an adjustable anchored single-incision mini-sling, Ajust®, with a standard mid-urethral sling, TVT-OTM: a health economic evaluation. BJU Int. 2013;112(8):1169-77.
29. Kilonzo M, Vale L, Stearns SC, Grant A, Cody J, Glazener CM, et al. Cost effectiveness of tension-free vaginal tape for the surgical management of female stress incontinence. Int J Technol Assess Health Care. 2004;20(4):455-63.
30. Valpas A, Rissanen P, Kujansuu E, Nilsson CG. A cost-effectiveness analysis of tension-free vaginal tape versus laparoscopic mesh colposuspension for primary female stress incontinence. Acta Obstet Gynecol Scand. 2006;85(12):1485-90.

For peer review only - http://bmjopen.bmj.com/site/about/guidelines.xhtml

Footnotes

Funding statement: This research was commissioned by the NIHR HTA Programme as project number 15/09/06. The views expressed are those of the authors and not necessarily those of the NHS, the NIHR or the Department of Health and Social Care, UK. The funders were not actively involved in the research process at any stage. The study design, collection, analysis and interpretation of data, the writing of the manuscript and the decision to submit it for publication were all performed independent of the funders.

Competing interests statement: None declared.

Patient consent for publication: Not required.

Author contributions: MJ, DC, EM, LT and LV designed the study. MJ and EM built the model and ran the analyses. DC, MB, LS, PM, JH, GM and LV helped to inform the model. All authors commented on the draft version of the manuscript and approved its final version.

Ethics approval and consent to participate: Not applicable.

Data availability statement: The authors declare that all of the data supporting the findings of this study are available within the article (or in the Supplementary Information).

For peer review only

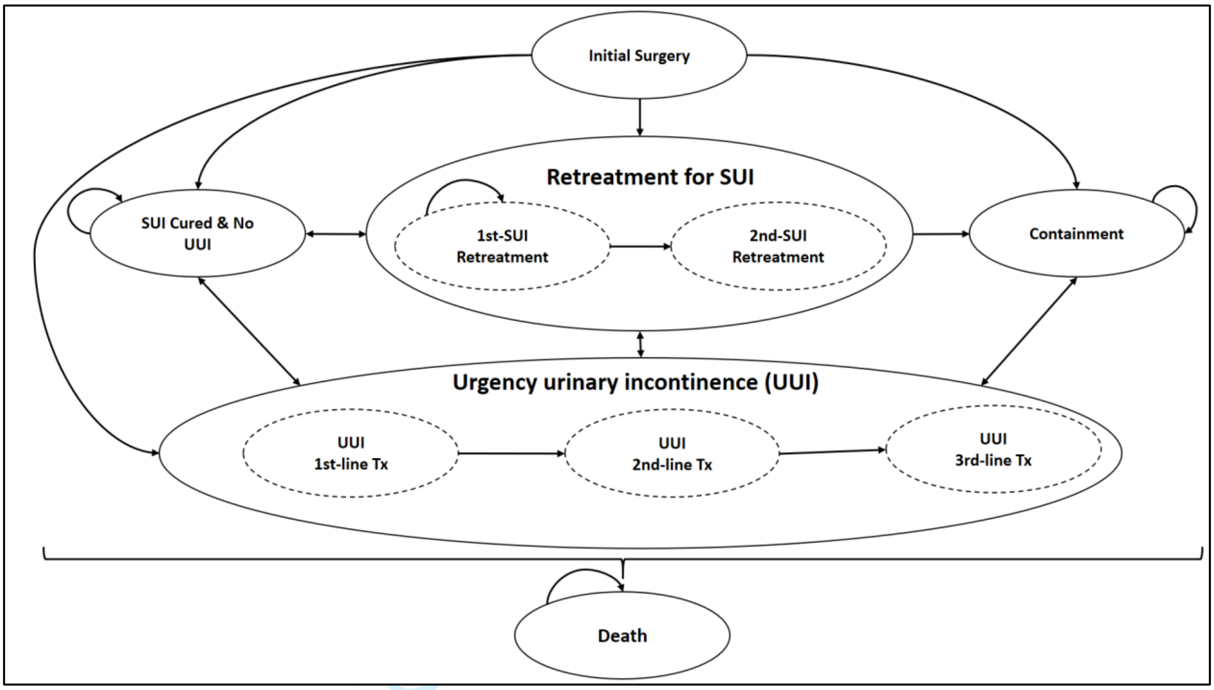

Figure 1 Markov model structure

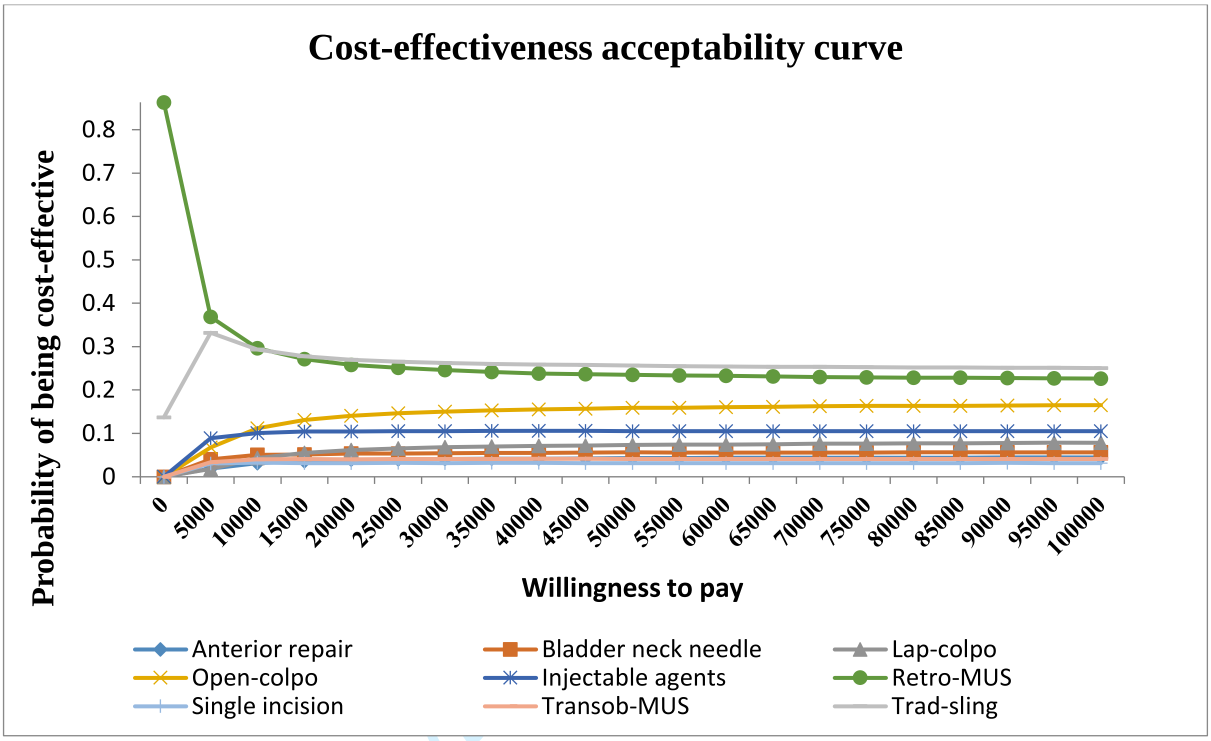

Figure 2 Cost-effectiveness acceptability curves for the nine surgical treatments: lifetime time horizon

Anterior repair: Anterior vaginal repair (anterior colporrhaphy); Bladder neck needle: Bladder neck needle suspensions; Lap-colpo: Laparoscopic retropubic colposuspension; Open-colpo: Open abdominal retropubic colposuspension; Trad-sling: Traditional suburethral retropubic sling procedures; MUS: Mid-urethral sling; Retro-MUS: Retropubic mid-urethral sling; Transob-MUS: Transobturator mid-urethral sling; Single incision: Single incision sling procedures ('mini-slings')

For peer review only

Table S1 Long-term cure rates after Retro-MUS used for extrapolation

Scenario	Time (years)	Cure rate	Source	Scale parameter (λ)	Shape parameter (γ)
Base-case analysis	1	84%	Meta-analysis	0.08799444	0.4932644
	2	78%			
Sensitivity analysis 1	1	85%	(1)	0.1969761	0.12368398
	2	80%			
Sensitivity analysis 2	1	78%	(2)	0.19391258	0.1788069
	13	67.5%			

For peer review only

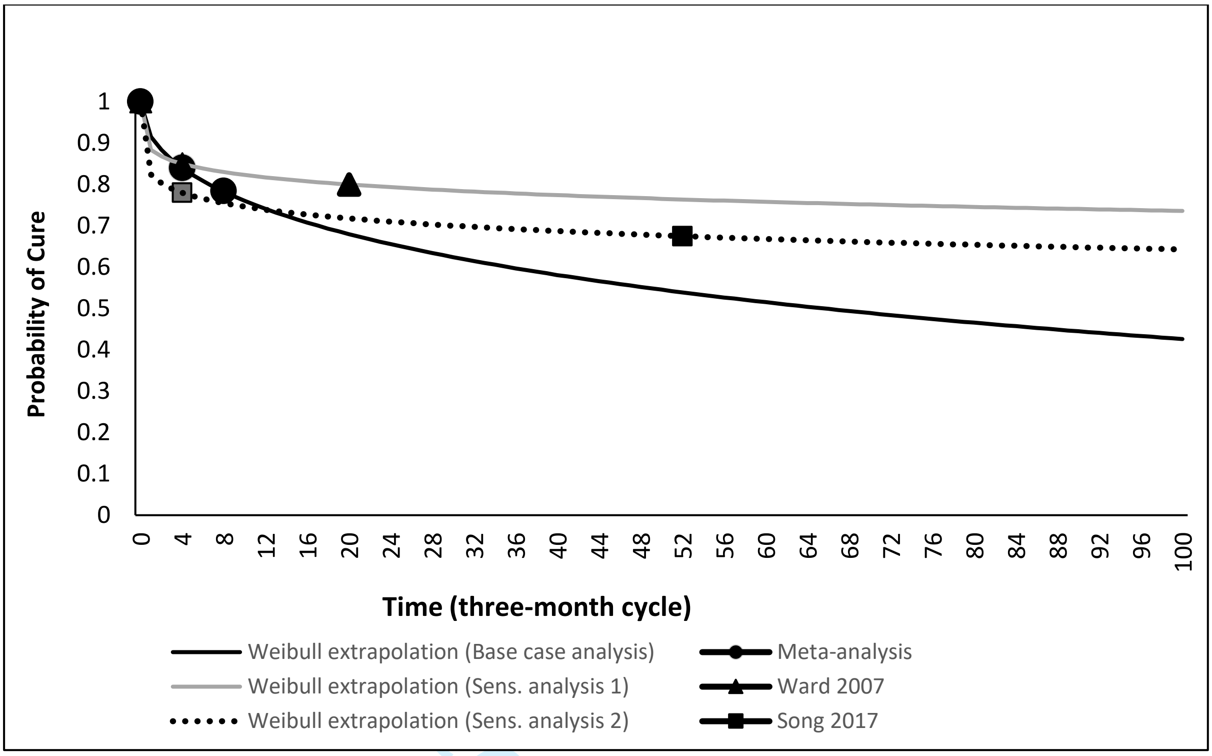

Figure S1 Extrapolated long-term cure rates after Retro-MUS surgery. Note: each cycle is 3 months long

Table S2 Weibull hazard function parameters for estimation of long term cure rates after Injectable agents

**Weibull Parameters**	**Cure rate**		
	Lower limit	Mean	Upper limit
**Lambda**	1.020173	0.767078	0.548895
**Gamma**	0.120006	0.137820	0.166837

For peer review only

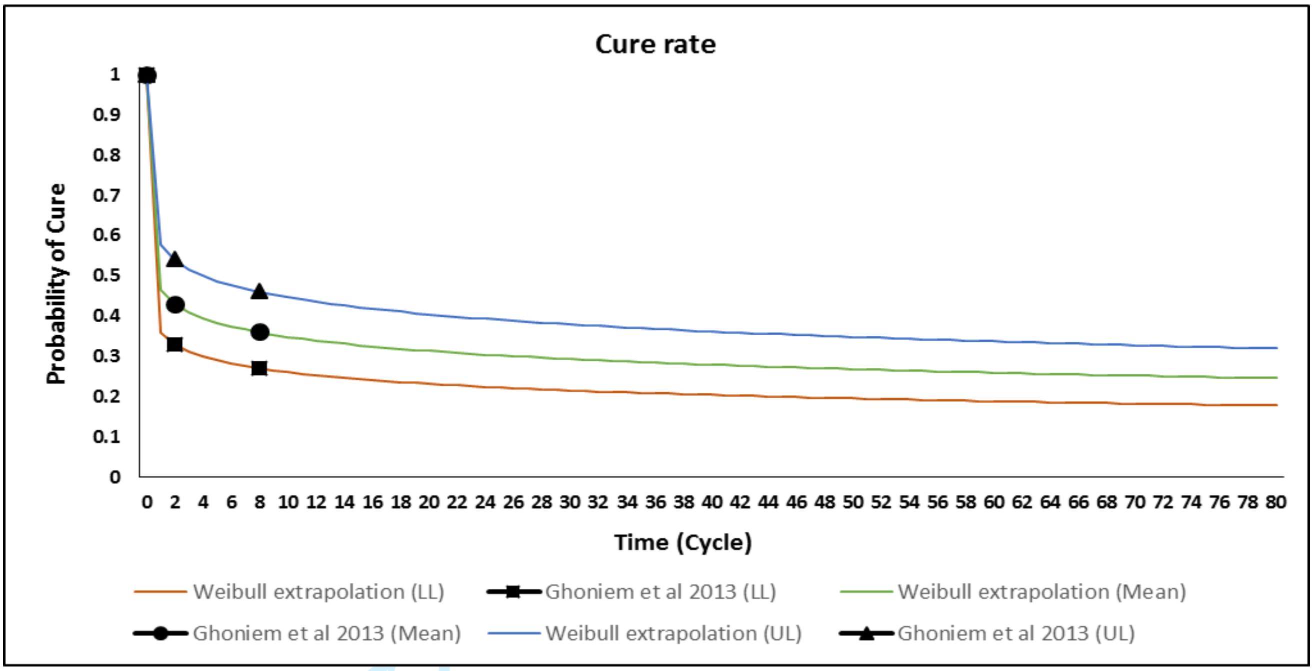

Figure S2 Extrapolated long-term cure rates after peri-urethral injections (Injectable agents). Note that each cycle is 3 months long

Table S3 Complications incidence rates after different surgical treatments (results from random-effect meta-analysis)

Complication	Infection		De novo symptoms of urgency or urgency incontinence		Voiding difficulties including urinary retention		Bladder or urethral perforation		Tape/mesh erosion / extrusion/exposure		Short term pain		Persistent pain or discomfort	
Surgery	Posterior Mean	Posterior SD	Posterior Mean	Posterior SD	Posterior Mean	Posterior SD	Posterior Mean	Posterior SD	Posterior Mean	Posterior SD	Posterior Mean	Posterior SD	Posterior Mean	Posterior SD
Anterior repair	NA	NA	15.84%	11.83%	0.01%	0.15%	NA	NA	NA	NA	NA	NA	NA	NA
Bladder neck needle	NA	NA	8.14%	4.87%	1.67%	2.61%	NA	NA	NA	NA	57.64%	30.08%	NA	NA
Open-colpo	24.33%	21.82%	5.78%	1.15%	3.00%	1.04%	1.02%	0.50%	0.0%	NA	20.94%	25.65%	0.15%	2.58%
Lap-colpo	NA	NA	8.38%	3.98%	6.32%	2.35%	2.10%	1.22%	0.0%	NA	NA	NA	NA	NA
Trad-sling	14.85%	12.49%	7.85%	3.23%	11.63%	3.60%	1.82%	1.11%	0.17%	0.34%	28.99%	28.12%	7.00%	8.98%
Retro-MUS	3.91%	4.72%	4.30%	0.66%	6.75%	0.95%	4.89%	0.50%	1.40%	0.31%	4.36%	1.14%	5.09%	2.37%
Transob-MUS	2.84%	1.03%	5.31%	0.51%	2.88%	0.44%	5.13%	0.50%	2.08%	0.26%	7.04%	2.08%	4.93%	1.22%
Single incision	3.22%	1.20%	6.47%	0.81%	2.15%	0.45%	0.37%	0.18%	2.38%	0.55%	4.54%	3.21%	1.28%	1.39%
Injectable agents	17.94%	24.59%	17.94%	24.59%	11.47%	20.70%	NA	NA	NA	NA	NA	NA	NA	NA

NA: Not available or applicable; Anterior repair: Anterior vaginal repair (anterior colporrhaphy); Bladder neck needle: Bladder neck needle suspensions; CrI: Creditable interval; Lap-colpo: Laparoscopic retropubic colposuspension; Open-colpo: Open abdominal retropubic colposuspension; Trad-sling: Traditional suburethral retropubic sling procedures; MUS: Mid-urethral sling; Retro-MUS: Retropubic mid-urethral sling; Transob-MUS: Transobturator mid-urethral sling; Single incision: Single incision sling procedures (‘mini-slings)

For peer review only

For peer review only

Table S4 Costs inputs

Resource use	Cost (£)	Source	HRG code
**Surgery cost**			
Anterior repair	2,236.50	NHS reference costs 2015/16 (1)	MA04C/D - Intermediate open, lower genital tract procedures-elective inpatient
Bladder neck needle	1,819.72	NHS reference costs 2015/16 (1)	LB26A/B - Intermediate endoscopic, prostate or bladder neck procedures-elective
Open-colpo	3,909.55	NHS reference costs 2015/16 (1)	LB59Z - Major, open or laparoscopic bladder neck procedures-elective inpatient
Lap-colpo	3,909.55	NHS reference costs 2015/16 (1)	LB59Z - Major, open or laparoscopic bladder neck procedures-elective inpatient
Trad-sling	1,924.32	NHS reference costs 2015/16 (1)	LB51A/B - Vaginal tape operations for urinary incontinence-elective inpatient
Retro-MUS	1,351.10	NHS reference costs 2015/16 (1)	LB51A/B - Vaginal tape operations for urinary incontinence-day case
Transob-MUS	1,351.10	NHS reference costs 2015/16 (1)	LB51A/B - Vaginal tape operations for urinary incontinence-day case
Single incision	1,040.54	Boyers et al. 2013 (2)	N/A
Injectable agents	1,820.29	Cody et al. 2003 (3)	N/A
Additional costs associated with surgery			
Incontinence pads	106.20 (3 month cost estimate)	NICE guidance document (4)	N/A
Urodynamic test	155	NHS reference costs 2015/16 (1)	N/A
Consultation pre-surgery (20 minute	46	PSSRU 2016 (5)	N/A
Urine dipstick analysis	3.94	NICE clinical guidelines (4)	N/A
Full blood count	6.31	NICE clinical guidelines (4)	N/A
Paracetamol 500mg (AAH) x 32	0.16 (daily cost based on a	BNF 2016 (6)	N/A
Lactulose 10g/15ml (AAH) x 10	0.50 (daily cost based on a	BNF 2016 (6)	N/A
**Urge incontinence treatment costs**			

Bladder training	97.81 (3 month cost estimate)	Appendices of NICE guidelines (4)	N/A
Band 6 hospital-based physiotherapist	45	PSSRU 2016 (5)	N/A
Oxybutynin 5mg (AAH) x 56	0.05 (daily cost based on a	BNF 2016 (6)	N/A
Consultant-led non-admitted follow-up	100	NHS reference costs 2015/16 (1)	N/A
Intermediate endoscopic bladder	971	NHS reference costs 2015/16 (1)	N/A
Botulinum toxin 100 unit powder for	138.20	BNF 2016 (6)	N/A
**Adverse event treatment costs**			
Co-amoxiclav 250/125mg (AAH) x 21	0.27 (daily cost based on a	BNF 2016 (6)	N/A
Attention to suprapubic bladder catheter	123	NHS reference costs 2015/16 (1)	N/A
Self-catheterisation	6.20 (daily cost based on 4	NICE guidance document (4)	N/A
Mesh excision or repair	1316.85	NHS reference costs 2015/16 (1)	N/A
Aspirin 300mg (AAH) x 28	0.40 (daily cost based on a	BNF 2016 (6)	N/A

For peer review only

For peer review only

Table S5 Utility values for different health states and utility decrement associated with complications

**Health status**	**Value**	**Source**	**Duration**
SUI pre-treatment	0.78	(1)	Variable
Mixed pre-treatment	0.78	(1)	Variable
Cured from SUI, and no urge urinary	0.85	(2)	Variable
Urge urinary incontinence	0.74	(2)	Variable
Pure SUI re-treatment	0.74	(2)	Variable
Mixed re-treatment	0.74	(2)	Variable
SUI failure of treatment (containment)	0.74	(2)	Variable
Mixed failure of treatment (containment)	0.74	(2)	Variable
**Utility decrement values for adverse events**			
Adverse event	**Utility decrement**	**Source**	**Duration**
Infection	- 0.19	(3)	2 weeks
Voiding difficulties (long-term)	- 0.23	(3)	3 months
Bladder/urethral perforation	- 0.08	(3)	2 weeks
Tape/mesh exposure/erosion	- 0.25	(3)	3 months
Short-term pain	-0.25	(3)	2 weeks
Persistent pain	- 0.25	(3)	6 months

For peer review only

Table S6 Results from deterministic sensitivity analyses-applying higher incidence rates of mesh complications after MUS procedures (lifetime time horizon)

Strategy	Cost (£)	Incremental cost (£)	QALY	Incremental QALY	ICER (£) (ΔCost/ΔQALY)	Probability cost-effective for different	
						£20,000	£30,000
Base-case analysis – estimating the incidence rate of mesh complications after MUS procedures based on the data from							
Retropubic MUS	8,099		24.22			25.8%	24.6%
Traditional sling	8,522	423	24.22	0.01	60,863	27.0%	26.2%
Urethral injection	9,554	1,032	23.86	-0.36	Dominated	10.5%	10.5%
Single incision sling	9,649	1,127	23.59	-0.63	Dominated	3.2%	3.2%
Transobturator	9,665	1,142	23.71	-0.51	Dominated	4.1%	4.1%
Bladder neck needle	10,125	1,603	23.69	-0.53	Dominated	5.4%	5.4%
Open-	10,977	2,455	24.10	-0.12	Dominated	14.1%	15.0%
Anterior vaginal	11,057	2,535	23.54	-0.69	Dominated	3.9%	4.1%
Laparoscopic-	11,797	3,274	23.83	-0.40	Dominated	6.2%	6.8%
SA – estimating the incidence rate of mesh complications for retropubic MUS (3.7%) and Transobturator MUS (2.8%)							
Retropubic MUS	8,138		24.20			24.5%	23.5%
Traditional sling	8,530	392	24.22	0.01	26,311	27.8%	26.8%
Urethral injection	9,560	1,030	23.85	-0.37	Dominated	10.7%	10.7%
Single incision sling	9,656	1,125	23.58	-0.63	Dominated	3.3%	3.2%
Transobturator	9,683	1,153	23.69	-0.53	Dominated	4.1%	4.0%
Bladder neck needle	10,131	1,601	23.67	-0.55	Dominated	5.9%	6.0%
Open-	10,978	2,448	24.09	-0.13	Dominated	14.1%	15.0%
Anterior vaginal	11,073	2,543	23.53	-0.69	Dominated	3.9%	4.2%
Laparoscopic-	11,805	3,275	23.81	-0.41	Dominated	5.9%	6.7%
SA –assuming 10% incidence rate of mesh complications after retropubic MUS and Transobturator MUS							
Retropubic MUS	8,238		24.19			23.9%	22.5%
Traditional sling	8,550	312	24.24	0.05	6,631	29.1%	28.1%
Urethral injection	9,596	1,045	23.86	-0.37	Dominated	11.2%	11.2%
Single incision sling	9,705	1,155	23.58	-0.65	Dominated	3.2%	3.1%
Transobturator	9,817	1,267	23.69	-0.55	Dominated	4.0%	4.2%
Bladder neck needle	10,180	1,629	23.69	-0.55	Dominated	5.8%	5.9%
Open-	11,010	2,460	24.08	-0.16	Dominated	13.1%	14.2%
Anterior vaginal	11,120	2,570	23.54	-0.70	Dominated	3.7%	4.1%
Laparoscopic-	11,843	3,292	23.84	-0.40	Dominated	6.0%	6.8%
SA –assuming 20% incidence rate of mesh complications after retropubic MUS and Transobturator MUS							
Retropubic MUS	8,381		24.17	0.00		23.6%	22.4%
Traditional sling	8,579	198	24.21	0.04	4,558	28.6%	27.6%
Urethral injection	9,644	1,064	23.86	-0.35	Dominated	10.7%	10.8%
Single incision sling	9,766	1,186	23.58	-0.64	Dominated	3.4%	3.3%
Transobturator	9,987	1,407	23.66	-0.56	Dominated	3.7%	3.8%
Bladder neck needle	10,237	1,657	23.67	-0.54	Dominated	5.6%	5.8%
Open-	11,048	2,469	24.07	-0.14	Dominated	14.1%	15.0%
Anterior vaginal	11,185	2,606	23.52	-0.69	Dominated	3.6%	3.8%
Laparoscopic-	11,892	3,312	23.82	-0.39	Dominated	6.7%	7.5%

For peer review only

Table S7 Results from deterministic sensitivity analyses-incorporating longer duration for persistent pain complication (lifetime time horizon)

Strategy	Cost (£)	Incremental cost (£)	QALY	Incremental QALY	ICER (£) (ΔCost/ΔQALY)	Probability cost-effective for different WTP thresholds (%)	
						£20,000	£30,000
Base-case analysis- assuming that persistent pain complication will last on average for 6 months							
Retropubic	8,099		24.22			25.8%	24.6%
Traditional sling	8,522	423	24.22	0.01	60,863	27.0%	26.2%
Urethral	9,554	1,032	23.86	-0.36	Dominated	10.5%	10.5%
Single incision	9,649	1,127	23.59	-0.63	Dominated	3.2%	3.2%
Transobturator	9,665	1,142	23.71	-0.51	Dominated	4.1%	4.1%
Bladder neck	10,125	1,603	23.69	-0.53	Dominated	5.4%	5.4%
Open-	10,977	2,455	24.10	-0.12	Dominated	14.1%	15.0%
Anterior vaginal	11,057	2,535	23.54	-0.69	Dominated	3.9%	4.1%
Laparoscopic-	11,797	3,274	23.83	-0.40	Dominated	6.2%	6.8%
SA – assuming that persistent pain complication will last on average for 36 months							
Retropubic	8,097		24.02			22.3%	20.9%
Traditional sling	8,523	426	24.01	-0.01	Dominated	23.7%	22.8%
Urethral	9,547	1,450	23.78	-0.24	Dominated	12.7%	12.6%
Single incision	9,646	1,549	23.45	-0.56	Dominated	3.4%	3.3%
Transobturator	9,665	1,567	23.50	-0.52	Dominated	3.2%	3.3%
Bladder neck	10,126	2,029	23.59	-0.43	Dominated	6.6%	6.7%
Open-	10,970	2,872	24.02	0.00	1,134,541	17.1%	18.2%
Anterior vaginal	11,060	90	23.43	-0.59	Dominated	4.0%	4.1%
Laparoscopic-	11,791	821	23.75	-0.27	Dominated	7.1%	8.1%
SA – assuming that persistent pain complication will last on average for 60 months							
Retropubic	8,103		23.90			20.8%	19.3%
Traditional sling	8,524	421	23.87	-0.03	Dominated	20.7%	19.9%
Urethral	9,550	1,447	23.74	-0.17	Dominated	13.8%	13.7%
Single incision	9,648	1,545	23.38	-0.52	Dominated	3.3%	3.2%
Transobturator	9,666	1,563	23.35	-0.56	Dominated	2.5%	2.6%
Bladder neck	10,127	2,024	23.54	-0.36	Dominated	7.1%	7.3%
Open-	10,976	2,872	23.99	0.09	33,380	19.4%	20.5%
Anterior vaginal	11,059	83	23.38	-0.61	Dominated	4.1%	4.4%
Laparoscopic-	11,793	818	23.71	-0.28	Dominated	8.3%	9.3%

For peer review only

Table S8 Results from deterministic sensitivity analyses-incorporating higher incidence rate for persistent pain complication after MUS procedures (lifetime time horizon)

Strategy	Cost (£)	Incremental cost (£)	QALY	Incremental QALY	ICER (£) (ΔCost/ΔQALY)	Probability cost-effective for different WTP thresholds (%)	
						£20,000	£30,000
Base-case analysis- assuming that incidence rate of persistent pain after retropubic MUS and Transobturator MUS are							
Retropubic MUS	8,099		24.22			25.8%	24.6%
Traditional sling	8,522	423	24.22	0.01	60,863	27.0%	26.2%
Urethral injection	9,554	1,032	23.86	-0.36	Dominated	10.5%	10.5%
Single incision sling	9,649	1,127	23.59	-0.63	Dominated	3.2%	3.2%
Transobturator MUS	9,665	1,142	23.71	-0.51	Dominated	4.1%	4.1%
Bladder neck needle	10,125	1,603	23.69	-0.53	Dominated	5.4%	5.4%
Open-	10,977	2,455	24.10	-0.12	Dominated	14.1%	15.0%
Anterior vaginal	11,057	2,535	23.54	-0.69	Dominated	3.9%	4.1%
Laparoscopic-	11,797	3,274	23.83	-0.40	Dominated	6.2%	6.8%
SA – assuming that incidence rate of persistent pain after retropubic MUS and Transobturator MUS is 10%							
Retropubic MUS	8,104		24.18			24.2%	23.0%
Traditional sling	8,523	419	24.20	0.03	15,067	28.4%	27.6%
Urethral injection	9,557	1,034	23.84	-0.37	Dominated	11.1%	11.1%
Single incision sling	9,649	1,126	23.57	-0.63	Dominated	3.2%	3.2%
Transobturator MUS	9,672	1,148	23.67	-0.54	Dominated	3.8%	3.8%
Bladder neck needle	10,128	1,605	23.66	-0.54	Dominated	5.7%	5.8%
Open-	10,977	2,454	24.07	-0.13	Dominated	13.8%	14.8%
Anterior vaginal	11,063	2,540	23.51	-0.69	Dominated	3.7%	3.9%
Laparoscopic-	11,799	3,275	23.81	-0.39	Dominated	6.2%	7.0%
SA – assuming that incidence rate of persistent pain after retropubic MUS and Transobturator MUS is 20%							
Retropubic MUS	8,109		24.13			23.3%	22.1%
Traditional sling	8,520	410	24.19	0.06	6,593	28.6%	27.5%
Urethral injection	9,557	1,037	23.83	-0.36	Dominated	10.8%	10.9%
Single incision sling	9,645	1,125	23.56	-0.63	Dominated	3.5%	3.5%
Transobturator MUS	9,677	1,158	23.61	-0.58	Dominated	3.8%	3.8%
Bladder neck needle	10,131	1,612	23.65	-0.54	Dominated	6.2%	6.3%
Open-	10,975	2,455	24.07	-0.12	Dominated	14.0%	15.1%
Anterior vaginal	11,063	2,543	23.49	-0.70	Dominated	3.5%	3.8%
Laparoscopic-	11,796	3,277	23.80	-0.39	Dominated	6.3%	7.1%

For peer review only

Table S9 Results from deterministic sensitivity analyses- applying higher incidence rates and longer duration for persistent pain after MUS procedures (lifetime time horizon)

Strategy	Cost (£)	Incremental cost (£)	QALY	Incremental QALY	ICER (£) (ΔCost/ΔQALY)	Probability cost-effective for different WTP thresholds (%)	
						**£20,000**	**£30,000**
Base-case analysis- assuming that incidence rate of persistent pain after retropubic MUS and Transobturator MUS are 5.09% and 4.93% and average duration of persistent pain is 6 months							
Retropubic MUS	8,099		24.22			25.8%	24.6%
Traditional sling	8,522	423	24.22	0.01	60,863	27.0%	26.2%
Urethral injection	9,554	1,032	23.86	-0.36	Dominated	10.5%	10.5%
Single incision sling	9,649	1,127	23.59	-0.63	Dominated	3.2%	3.2%
Transobturator MUS	9,665	1,142	23.71	-0.51	Dominated	4.1%	4.1%
Bladder neck needle	10,125	1,603	23.69	-0.53	Dominated	5.4%	5.4%
Open-	10,977	2,455	24.10	-0.12	Dominated	14.1%	15.0%
Anterior vaginal	11,057	2,535	23.54	-0.69	Dominated	3.9%	4.1%
Laparoscopic-	11,797	3,274	23.83	-0.40	Dominated	6.2%	6.8%
SA – assuming that incidence rate of persistent pain after retropubic MUS and Transobturator MUS are 20% and average duration of persistent pain is 60 months							
Retropubic MUS	8,118		22.97			4.2%	3.8%
Traditional sling	8,527	409	23.63	0.66	619	30.8%	29.5%
Urethral injection	9,560	1,033	23.37	-0.26	Dominated	17.0%	16.6%
Single incision sling	9,652	1,125	22.90	-0.72	Dominated	2.8%	2.7%
Transobturator MUS	9,683	1,156	22.25	-1.38	Dominated	0.1%	0.1%
Bladder neck needle	10,130	1,604	23.11	-0.52	Dominated	7.0%	7.0%
Open-	10,978	2,451	23.68	0.05	46,732	25.8%	27.0%
Anterior vaginal	11,064	86	22.89	-0.79	Dominated	3.4%	3.5%
Laparoscopic-	11,797	819	23.32	-0.36	Dominated	9.0%	9.9%

For peer review only

Table S10 **Results from deterministic sensitivity analyses-applying different values for short and long term cure rates after retropubic MUS (lifetime time horizon)**

Strategy	Cost (£)	Incremental cost (£)	QALY	Incremental QALY	ICER (£) (ΔCost/ΔQALY)	Probability cost-effective for different	
						£20,000	£30,000
Base-case analysis – applying estimated mean values from the meta-analysis for short and long term cure rates after retropubic MUS							
Retropubic MUS	8,099		24.22			25.8%	24.6%
Traditional sling	8,522	423	24.22	0.01	60,863	27.0%	26.2%
Urethral injection therapy	9,554	1,032	23.86	-0.36	Dominated	10.5%	10.5%
Single incision sling	9,649	1,127	23.59	-0.63	Dominated	3.2%	3.2%
Transobturator MUS	9,665	1,142	23.71	-0.51	Dominated	4.1%	4.1%
Bladder neck needle	10,125	1,603	23.69	-0.53	Dominated	5.4%	5.4%
Open-colposuspension	10,977	2,455	24.10	-0.12	Dominated	14.1%	15.0%
Anterior vaginal repair	11,057	2,535	23.54	-0.69	Dominated	3.9%	4.1%
Laparoscopic-	11,797	3,274	23.83	-0.40	Dominated	6.2%	6.8%
SA – applying values from Ward and colleagues,(1) for short and long term cure rates after retropubic MUS							
Retropubic MUS	7,163		24.55			45.7%	43.3%
Traditional sling	8,493	1,330	24.22	-0.33	Dominated	16.6%	16.7%
Urethral injection therapy	9,418	2,255	23.89	-0.66	Dominated	8.3%	8.5%
Transobturator MUS	9,422	2,260	23.77	-0.77	Dominated	4.2%	4.3%
Single incision sling	9,429	2,267	23.65	-0.90	Dominated	3.3%	3.2%
Bladder neck needle	9,944	2,782	23.72	-0.82	Dominated	4.6%	4.9%
Anterior vaginal repair	10,855	3,692	23.58	-0.97	Dominated	3.5%	3.8%
Open-colposuspension	10,878	3,715	24.11	-0.44	Dominated	9.0%	9.9%
Laparoscopic-	11,653	4,490	23.86	-0.69	Dominated	4.9%	5.6%
SA – applying data from Song and colleagues,(2) for short and long term cure rates after retropubic MUS							
Retropubic MUS	7,394		24.49			42.1%	39.9%
Traditional sling	8,522	1,128	24.22	-0.27	Dominated	19.3%	19.0%
Urethral injection therapy	9,437	2,043	23.90	-0.59	Dominated	8.1%	8.3%
Single incision sling	9,464	2,070	23.64	-0.85	Dominated	3.1%	3.2%
Transobturator MUS	9,475	2,080	23.77	-0.72	Dominated	4.1%	4.2%
Bladder neck needle	9,979	2,585	23.73	-0.76	Dominated	5.3%	5.4%
Anterior vaginal repair	10,881	3,486	23.59	-0.90	Dominated	3.6%	3.9%
Open-colposuspension	10,916	3,522	24.11	-0.38	Dominated	9.8%	10.7%
Laparoscopic-	11,680	4,286	23.85	-0.64	Dominated	4.6%	5.3%

For peer review only

For peer review only

References for online supplementary files

1. Ward KL, Hilton P. Tension-free vaginal tape versus colposuspension for primary urodynamic stress incontinence: 5-year follow up. BJOG : an international journal of obstetrics and gynaecology. 2008;115(2):226-33.
2. Song PH, Kwon DH, Ko YH, Jung HC. The Long-Term Outcomes of the Tension-free Vaginal Tape Procedure for Treatment of Female Stress Urinary Incontinence: Data from Minimum 13 Years of Follow-Up. LUTS: Lower Urinary Tract Symptoms. 2017;9(1):10-4.
3. Department of Health. Reference Costs 2015-162016.
4. Boyers D, Kilonzo M, Mostafa A, Abdel-Fattah M. Comparison of an adjustable anchored single-incision mini-sling, Ajust®, with a standard mid-urethral sling, TVT-OTM: a health economic evaluation. BJU Int. 2013;112(8):1169-77.
5. Cody J. Systematic review of the clinical effectiveness and cost-effectiveness of tension-free vaginal tape for treatment of urinary stress incontinence. 2003.
6. Urinary incontinence in women: management Clinical guideline [CG171] 2015. Available from: https://www.nice.org.uk/guidance/cg171.
7. Curtis L, Burns A. Unit Costs of Health & Social Care 2016: The University of Kent; 2016.
8. British National Formulary. BNF Online [02 March 2017]. Available from: https://www.medicinescomplete.com/mc/?utm_source=bnforg&utm_medium=homepage&utm_campaign=medicinescomplete.
9. Manca A, Sculpher MJ, Ward K, Hilton P. A cost-utility analysis of tension-free vaginal tape versus colposuspension for primary urodynamic stress incontinence. BJOG : an international journal of obstetrics and gynaecology. 2003;110(3):255-62.
10. Haywood KL, Garratt AM, Lall R, Smith JF, Lamb SE. EuroQol EQ-5D and condition-specific measures of health outcome in women with urinary incontinence: reliability, validity and responsiveness. Qual Life Res. 2008;17(3):475-83.
11. Shepherd JP, Lowder JL, Jones KA, Smith KJ. Retropubic and transobturator midurethral slings: a decision analysis to compare outcomes including efficacy and complications. Int Urogynecol J Pelvic Floor Dysfunct. 2010;21(7):787-93.

For peer review only

BMJ Open

Economic Evaluation of Surgical Treatments for Women with Stress Urinary Incontinence: A Cost-Utility and Value of Information Analysis

Journal:	*BMJ Open*
Manuscript ID	bmjopen-2019-035555.R1
Article Type:	Original research
Date Submitted by the Author:	06-Feb-2020
Complete List of Authors:	Javanbakht, Mehdi; Institute of Health & Society, Newcastle University, Health Economics ; Institute of Health & Society, Newcastle University<br>Moloney, Eoin; Institute of Health & Society, Newcastle University, Health Economics Group, Institute of Health and Society<br>Brazzelli, Miriam; University of Aberdeen, Health Services Research Unit<br>Wallace, Sheila<br>Ternent, Laura; Newcastle University, Institute of Health & Society<br>Omar, Muhammad; University of Aberdeen, Academic Urology Unit/ Cochrane Incontinence Group<br>Monga, Ash ; University of Southampton<br>Saraswat, L; University of Aberdeen,<br>Mackie, Phil<br>Becker, Frauke; University of Oxford, Health Economics Research Centre<br>Imamura, Mari; University of Aberdeen<br>Hudson, Jemma; The Centre for Healthcare Randomised Trials (CHaRT), Health Services Research Unit, University of Aberdeen, Foresterhill<br>Shimonovich, Michal ; University of Aberdeen<br>MacLennan, Graeme; University of Aberdeen, Health Services Research Unit<br>Vale, Luke; Newcastle University, Health Economics Group, Institute of Health and Society<br>Craig, Dawn; Institute of Health & Society, Newcastle University, Health Economics Group, Institute of Health and Society
**Primary Subject Heading**:	Surgery
Secondary Subject Heading:	Health economics
Keywords:	Health economics < HEALTH SERVICES ADMINISTRATION & MANAGEMENT, Urinary incontinences < UROLOGY, Adult surgery < SURGERY

SCHOLARONE™ Manuscripts

I, the Submitting Author has the right to grant and does grant on behalf of all authors of the Work (as defined in the below author licence), an exclusive licence and/or a non-exclusive licence for contributions from authors who are: i) UK Crown employees; ii) where BMJ has agreed a CC-BY licence shall apply, and/or iii) in accordance with the terms applicable for US Federal Government officers or employees acting as part of their official duties; on a worldwide, perpetual, irrevocable, royalty-free basis to BMJ Publishing Group Ltd ("BMJ") its licensees and where the relevant Journal is co-owned by BMJ to the co-owners of the Journal, to publish the Work in this journal and any other BMJ products and to exploit all rights, as set out in our licence.

The Submitting Author accepts and understands that any supply made under these terms is made by BMJ to the Submitting Author unless you are acting as an employee on behalf of your employer or a postgraduate student of an affiliated institution which is paying any applicable article publishing charge ("APC") for Open Access articles. Where the Submitting Author wishes to make the Work available on an Open Access basis (and intends to pay the relevant APC), the terms of reuse of such Open Access shall be governed by a Creative Commons licence – details of these licences and which Creative Commons licence will apply to this Work are set out in our licence referred to above.

Other than as permitted in any relevant BMJ Author's Self Archiving Policies, I confirm this Work has not been accepted for publication elsewhere, is not being considered for publication elsewhere and does not duplicate material already published. I confirm all authors consent to publication of this Work and authorise the granting of this licence.

For peer review only

Economic Evaluation of Surgical Treatments for Women with Stress Urinary Incontinence: A Cost-Utility and Value of Information Analysis

Running title: Cost-Utility of Surgical Treatments for Stress Urinary Incontinence

Mehdi Javanbakht[1], Eoin Moloney[1*], Miriam Brazzelli[2], Sheila Wallace[1], Laura Ternent[1], Muhammad Imran Omar[3], Ash Monga[4], Lucky Saraswat[5], Phil Mackie[6], Frauke Becker[1], Mari Imamura[2], Jemma Hudson[2], Michal Shimonovich[2], Graeme MacLennan[2], Luke Vale[1], Dawn Craig[1]

1- Institute of Health & Society, Newcastle University, Newcastle upon Tyne, UK
2- Health Services Research Unit, University of Aberdeen, Aberdeen, UK
3- Guidelines Office Methodology Supervisor, European Association of Urology, Arnhem, Netherlands
4- University Hospitals Southampton Foundation Trust, Southampton, UK
5- Aberdeen Royal Infirmary, Aberdeen, UK
6- Scottish Public Health Network, Head of Knowledge and Research Services, NHS Health Scotland

***Corresponding author:** Eoin Moloney
Institute of Health & Society, Baddiley-Clark Building, Newcastle University, Richardson Road, Newcastle upon Tyne, NE2 4AX; E-mail: eoin.moloney@newcastle.ac.uk; Telephone: +44 (0) 191 208 7359

Word count: abstract: 295; Manuscript: Approx. 3,000

For peer review only - http://bmjopen.bmj.com/site/about/guidelines.xhtml

Abstract

Objectives: Stress urinary incontinence (SUI) and stress-predominant mixed urinary incontinence (MUI) are common conditions that can have a negative impact on the quality of life of patients and serious cost implications for health care providers. The objective of this study was to assess the cost-effectiveness of nine different surgical interventions for treatment of SUI and stress-predominant MUI from an NHS and personal social services perspective in the UK.

Methods: A Markov microsimulation model was developed to compare the costs and effectiveness of nine surgical interventions. The model was informed by undertaking a systematic review of clinical effectiveness, and network meta-analysis. The main clinical parameters in the model were the cure and incidence rates of complications after different interventions. The outcomes from the model were expressed in terms of cost per quality-adjusted life-years (QALYs) gained. In addition, expected value of perfect information (EVPI) analyses were conducted to quantify the main uncertainties facing decision-makers.

Results: The base-case results suggest that Retropubic mid-urethral sling (Retro-MUS) is the most cost-effective surgical intervention over a 10-year and lifetime time horizon. The probabilistic results show that Retro-MUS and traditional sling are the interventions with the highest probability of being cost-effective across all willingness-to-pay (WTP) thresholds over a lifetime time horizon. The value of information analysis results suggest that the largest value appears to be in removing uncertainty around the incidence rates of complications, the relative treatment effectiveness, and health utility values.

Conclusions: Although Retro-MUS appears, at this stage, to be a cost-effective intervention, research is needed on possible long-term complications of all surgical treatments to provide reassurance of safety, or earlier warning of unanticipated adverse effects. The value of information analysis supports the need, as a first step, for further research to improve our knowledge of the actual incidence of complications.

Key Words: Stress urinary incontinence, Surgery, Economic evaluation, Cost-effectiveness analysis, Markov microsimulation.

For peer review only - http://bmjopen.bmj.com/site/about/guidelines.xhtml

Article Summary

Strengths and limitations of this study

- An economic model has been developed to consider the relative cost-effectiveness of nine different surgical interventions for stress urinary incontinence and stress-predominant mixed urinary incontinence in a UK setting.
- The model has been informed and populated based on a comprehensive systematic review and network meta-analysis of the clinical effectiveness of surgical interventions.
- Uncertainty in the model results has been explored through a probabilistic sensitivity analysis, and value of information analysis to consider the value of future research.
- Due to limited long-term data on effectiveness, short-term data were extrapolated over a longer-term time horizon.

For peer review only

For peer review only - http://bmjopen.bmj.com/site/about/guidelines.xhtml

Introduction

Stress urinary incontinence (SUI) in women is a disabling and common condition which reduces quality of life and causes significant stress and concern amongst individual patients (1, 2). Commonly defined as the 'involuntary leakage of urine' due to activities such as coughing, sneezing or exercising, the prevalence of SUI varies (20 to 50%) but is greater in women who have had children and in older women (3, 4). Associated conditions include urge urinary incontinence (UUI), which is characterised by a sudden and uncontrollable need to urinate (i.e. urgency), and mixed urinary incontinence (MUI) which refers to a combination of the symptoms associated with SUI and UUI (2). Where the symptoms of SUI dominate those of UUI in a patient with MUI, stress-predominant MUI is said to exist.

In addition to the emotional impact that SUI can have on patients, there are serious cost implications associated with the condition, both for the health care provider and the individual patient (5). Amongst the previous studies to have investigated the economic burden of the condition, one published study based on data from three European countries suggested that the total cost of SUI is approximately £818 million in the UK (2), whilst another suggested a health care cost to the UK NHS of £117 million per year (SUI only) (5). In addition to the health service costs, patients may incur out-of-pocket expenses due to the need to purchase consumables and services not available through their health care provider (5).

Conservative treatment with physiotherapy to deliver pelvic floor muscle exercises and bladder training is the first line of treatment for patients with SUI. However, when this fails, or when insufficient relief from symptoms is achieved, surgery is the recommended treatment (6). The aim of surgery is to support or partially obstruct the bladder neck and/or urethra, thus preventing the leakage of urine on exertion (7). Surgical techniques have evolved over time, moving most recently to more minimally invasive techniques, such as mid-urethral slings (MUS) where a synthetic mesh or tape is placed under the urethra and secured using a number of different methods (8). However, it is unclear if these newly available treatments such as retropubic mid-urethral sling (Retro-MUS), single incision sling and injectable bulking agents really result in equivalent or better cost and health outcomes than older operations that were previously available (such as anterior vaginal repair or colposuspension).

The wide range of surgical operations available, the different techniques used to perform these operations, and the lack of a consensus amongst surgeons regarding which approach to use, make it challenging to establish which procedure should be used to treat SUI or stress-predominant MUI. Health economic techniques have become increasingly important as a tool to help inform decisions between treatments and interventions. They allow for the consideration of all relevant costs (from the specified perspective) and clinical outcomes associated with the treatments being compared. Findings can then be used to make a decision on how best to allocate resources in the most cost-effective way. This study was conducted as part of a larger project to evaluate the cost-effectiveness of Retro-MUS versus eight different comparator surgical interventions for the treatment of women with SUI or stress-predominant MUI (9).

Methods

Study Design

A Markov microsimulation (MM) model was developed to assess cost-effectiveness. The model was informed by a review of published economic evaluations, and the findings of a systematic review of clinical effectiveness and network meta-analyses (including 120 trials) conducted as part of the larger project to estimate the relative effectiveness of nine different surgical treatments (9). Retro-MUS was chosen as the intervention against which all other surgical interventions would be compared, due to the fact that it is the most common type of surgery and it is widely regarded as standard practice (10). The eight comparators were: 1) Anterior vaginal repair or anterior colporrhaphy (Anterior repair), 2) Bladder neck needle suspensions (Bladder neck needle), 3) Open abdominal retropubic colposuspension (Open-colpo), 4) Laparoscopic retropubic colposuspension (Lap-colpo), 5) Traditional sub-urethral retropubic

For peer review only - http://bmjopen.bmj.com/site/about/guidelines.xhtml

sling procedures (Trad-sling), 6) Transobturator mid-urethral sling (Transob-MUS), 7) Single incision sling procedures (Single incision sling) and 8) Peri-urethral injection bulking agents (Injectable agents).

Health outcomes from the model were expressed in terms of quality-adjusted life-years (QALYs) and costs in 2018/19 GBP (£). The costs were estimated from the NHS and personal social services perspective. Both costs and QALYs were evaluated over 1-year, 10-year and lifetime time horizons and discounted using a 3.5% annual discount rate (11). The expected cost and QALYs for each of the strategies were compared using incremental cost-effectiveness ratios (ICERs) where appropriate.

Target Population

The model was based on a hypothetical group of women (45-55 years) with SUI or stress-predominant MUI, undergoing surgical intervention for their condition.

Model Structure

The model structure is presented in Figure 1. Treatment history for both SUI/stress-predominant MUI and UUI was recorded for each modeled woman and used to define the patient transitions to different health states and treatment types. The model assumes that patients can receive a maximum of three surgical treatments for treatment of SUI/stress-predominant MUI, which includes the initial surgery plus two subsequent re-treatments. MUS can be offered after the failure of all surgery types, and therefore re-treatment was always assumed to be either Retro-MUS (55%) or Transob-MUS (45%). If all three surgeries fail, patients have to manage their symptoms using containment products. The model allows for individuals to elect to use containment products at any point after the initial treatment has failed. Patients with stress-predominant MUI who still have UUI after SUI is cured, or those who develop UUI due to a surgery, i.e. as a complication following the procedure, will receive three lines of treatment based on clinical guidelines including first-line (Bladder training), second-line (Medication) and third-line treatment (Botulinum toxin A). The model was developed in TreeAge Pro® package (TreeAge Software, Inc., Williamstown, MA, USA) (12).

Figure 1 Markov model structure

Model Inputs

Effectiveness of surgical treatments

The relative effectiveness of surgical treatments, in terms of subjective cure rates, were based on the results of a network meta-analysis (9). Table 1 describes the mean and median odds ratios (ORs) for different surgical treatments vs Retro-MUS. The absolute cure rates were calculated in the model by combining the information on relative cure rates described in Table 1 with the absolute cure rates for Retro-MUS, presented in Table 2. The long-term recurrence rates after Retro-MUS were estimated using parametric survival models. Scale and shape parameters were estimated for a Weibull hazard function using the reported cure rates at 1 and 5 years (Table S1, Figure S1, Table S2, and Figure S2, respectively). In the base-case analysis, it was assumed that 75% of women whose first treatment was not successful would seek re-treatment and 30% of women whose first retreatment failed would seek a second retreatment (13). Due to data limitations, it was not possible to estimate the success rates of re-treatment following failure of prior surgery. Therefore, in the model it was assumed that subsequent surgeries (Retro-MUS or Transob-MUS) were 90% as effective as when administered as a primary surgery.

For peer review only - http://bmjopen.bmj.com/site/about/guidelines.xhtml

Table 1 Odds ratios of cure rates for surgical interventions compared with Retro-MUS

Treatment	Mean	Median	Lower bound of 95% CrI	Upper bound of 95% CrI
Transob-MUS vs Retro-MUS	0.742	0.738	0.588	0.923
Open-colpo vs Retro-MUS	0.874	0.853	0.544	1.325
Lap-colpo vs Retro-MUS	0.605	0.58	0.315	1.046
Trad-sling vs Retro-MUS	1.106	1.061	0.623	1.846
Single incision vs Retro-MUS	0.511	0.504	0.36	0.699
Bladder neck needle vs Retro-MUS	0.368	0.34	0.154	0.745
Anterior repair vs Retro-MUS	0.235	0.22	0.105	0.452

Anterior repair: Anterior vaginal repair (anterior colporrhaphy); Bladder neck needle: Bladder neck needle suspensions; CrI: Credible interval; Lap-colpo: Laparoscopic retropubic colposuspension; Open-colpo: Open abdominal retropubic colposuspension; Trad-sling: Traditional suburethral retropubic sling procedures; MUS: Mid-urethral sling; Retro-MUS: Retropubic mid-urethral sling; Transob-MUS: Transobturator mid-urethral sling; Single incision: Single incision sling procedures ('mini-slings')

Table 2 Estimation of absolute cure rates after Retro-MUS in different time points-results from meta-analysis

Time	Median	95% CrI	Number of studies	Number of participants
6 months	0.776	(0.175, 0.983)	17	908
12 months	0.841	(0.214, 0.990)	44	2882
24 months	0.784	(0.454, 0.941)	6	315
36 months	0.341	(0.001, 0.995)	5	205
60 months	0.329	(0.005, 0.979)	3	377

Complication and mortality rates after surgical treatments

The model incorporated only severe complications/adverse events that are most important in terms of their effect on patient's quality of life, cost and duration of impact. To estimate complication incidence rates, random-effects meta-analysis models were fitted to data on complication rates from the RCTs identified within the wider systematic review (14). All of the complications included in the model, and estimated incidence rates, are presented in Table S3. In the model, these complication rates were defined as distributions (Beta distribution). Where evidence for particular adverse events associated with specific procedures were not identified, it was assumed that this complication would not occur. Age-specific all-cause mortality rates were derived from general population mortality statistics reported in national life tables (15). The effectiveness of the three lines of treatments for UUI were informed by the results from previous meta-analyses (6, 16).

Resource use and unit costs

Surgeries for SUI vary in terms of the complexity of the procedure and the setting in which surgery is conducted. For patients undergoing Anterior repair, Bladder neck needle, Open-colpo, Lap-colpo, and Trad-sling, surgery would typically be conducted in an inpatient setting. Patients undergoing Retro-MUS, Transob-MUS, Single incision sling and Injectable agents would typically be treated in a day-case setting. In addition to the cost of individual surgeries, costs associated with complementary tests,

For peer review only - http://bmjopen.bmj.com/site/about/guidelines.xhtml

treatments and consultations that would typically be carried out in advance of, and following, each surgery were also considered. Unit costs were derived from UK NHS reference costs (17), Personal Social Services Research Unit (PSSRU) (18) and British National Formulary (BNF) (19) for medication. The price year of the analysis was 2018/19. Where sources used to derive costs were from prior to 2019, these costs were inflated to a 2019 price year. All costs are reported in Table S4.

Health Utility

The baseline health utility value for pre-treatment SUI was derived from a previous UK-based economic evaluation comparing two types of surgical intervention for this condition (20). The utility value for a successful treatment was derived from a UK-based study exploring health outcomes in women with urinary incontinence (21). Utility decrements associated with each complication included in the model were obtained from previous studies (22, 23) (Table S5).

Patient and Public Involvement

As part of the larger project (9), patient and public involvement (PPI) was included to understand patient's thoughts and feelings regarding their condition and treatment and to help add context to the review and meta-analysis of clinical effectiveness. However, no additional PPI was sought for this modelling work.

Analysis and Sensitivity analyses

Both deterministic and probabilistic sensitivity analyses (PSA) were used to explore parameter and other forms of uncertainty surrounding the estimates of cost-effectiveness. Deterministic sensitivity analyses including; applying higher incidence rates of mesh complications, longer durations of persistent pain complication, higher incidence rates for persistent pain complication post MUS procedures, and applying different values for short and long-term cure rates after retropubic MUS were performed to determine the impact of changing key parameters and assumptions on the model results. The PSA was run with 1,000 simulations for each patient, and cost-effectiveness acceptability curves (CEACs) were produced in order to identify the probability of the different surgeries being cost-effective across a range of willingness-to-pay (WTP) thresholds. The economic model was used to quantify the main uncertainties facing decision-makers and to help inform decisions about the direction of future research. This was explored through value of information (VOI) analysis methods: expected value of perfect information (EVPI) and expected value of partial perfect information (EVPPI) analysis. In total, it was assumed that in the UK 15,000 surgical treatments are conducted annually for the treatment of SUI.

Results

Base-case analysis

The base-case analysis results are presented in Table 3, and in the form of a CEAC (lifetime time horizon) in Figure 2. The table reports strategies from the least to the most costly. Over a lifetime time horizon, Retro-MUS is, on average, the least costly (£8,666) and the second most effective (24.005 QALYs) surgical treatment. Trad-sling is more costly (+ £405) but also more effective (+ 0.009) than Retro-MUS over the lifetime, however it is not cost-effective, based on the NICE WTP threshold, with an ICER of £45,340. All other surgical treatments are dominated, as they are more costly and less effective than Retro-MUS. Both Retro-MUS and Trad-sling have similarly high probabilities of being cost-effective at £20,000 (51% and 43%, respectively) and £30,000 (48% and 45%, respectively) WTP thresholds over a lifetime time horizon. Over a 10-year time horizon, Retro-MUS is the dominant strategy with a greater than 90% probability of being cost-effective at the £20,000 and £30,000 WTP thresholds presented. However, over a 1-year time horizon Single incision sling is dominant compared to all other strategies, except for Anterior repair and Open-colpo, which are both marginally more effective.

For peer review only - http://bmjopen.bmj.com/site/about/guidelines.xhtml

Figure 2 shows that over a lifetime time horizon, both Trad-sling and Retro-MUS have a high probability of being cost-effective at all WTP threshold values presented. Given the number of comparators, if the interventions were comparable we would expect an 11% chance of each being cost-effective. The only other strategy with a reasonably sized probability of being cost-effective over the lifetime is Open-colpo (6% and 7% probabilities of being cost-effective at £20,000 and £30,000 WTP thresholds, respectively). No other strategy has a significant probability of being cost-effective at any of the WTP thresholds presented.

Table 3 Results of the probabilistic analysis 1-, 10 years and lifetime time horizons

Time horizon	Strategy	Cost (£)	Incremental cost (£)	QALY	Incremental QALY	ICER (£) (ΔCost/ΔQALY)	Probability of being cost-effective at different threshold (%)	
							£20,000	£30,000
1 Year	Single incision sling	1,844		0.764			100%	100%
	Transob-MUS	2,470	626	0.752	-0.012	Dominated	0%	0%
	Retro-MUS	2,490	646	0.752	-0.012	Dominated	0%	0%
	Bladder neck needle	2,682	838	0.757	-0.006	Dominated	0%	0%
	Injectable agents	2,705	861	0.742	-0.021	Dominated	0%	0%
	Trad-sling	2,941	1,097	0.725	-0.038	Dominated	0%	0%
	Anterior repair	2,955	1,111	0.766	0.002	522,756	0%	0%
	Open-colpo	4,847	1,891	0.775	0.009	212,116	0%	0%
	Lap-colpo	4,875	28	0.765	-0.010	Dominated	0%	0%
10 Years	Retro-MUS	4,905		7.270			95.0%	93.0%
	Single incision sling	5,271	366	7.023	-0.248	Dominated	0%	0%
	Trad-sling	5,471	566	7.215	-0.056	Dominated	5.0%	7.0%
	Transob-MUS	5,519	614	7.107	-0.163	Dominated	0%	0%
	Injectable agents	5,882	977	7.068	-0.202	Dominated	0%	0%
	Bladder neck needle	6,101	1,197	7.025	-0.245	Dominated	0%	0%
	Anterior repair	6,629	1,725	6.967	-0.303	Dominated	0%	0%
	Open-colpo	7,596	2,691	7.204	-0.066	Dominated	0%	0%
	Lap-colpo	7,997	3,092	7.106	-0.164	Dominated	0%	0%
Lifetime	Retro-MUS	8,666		24.005			51.0%	48.0%
	Trad-sling	9,071	405	24.014	0.009	45,340	43.0%	45.0%
	Transob-MUS	10,174	1,103	23.435	-0.580	Dominated	0%	0%
	Single incision sling	10,189	1,118	23.221	-0.793	Dominated	0%	0%
	Injectable agents	10,292	1,221	23.512	-0.503	Dominated	0%	0%
	Bladder neck needle	10,803	1,732	23.312	-0.702	Dominated	0%	0%
	Open-colpo	11,605	2,535	23.839	-0.175	Dominated	6.0%	7.0%
	Anterior repair	11,609	2,539	23.168	-0.847	Dominated	0%	0%
	Lap-colpo	12,440	3,369	23.522	-0.492	Dominated	0%	0%

Anterior repair: Anterior vaginal repair (anterior colporrhaphy); Bladder neck needle: Bladder neck needle suspensions; CrI: Credible interval; Lap-colpo: Laparoscopic retropubic colposuspension; Open-colpo: Open abdominal retropubic colposuspension; Trad-sling: Traditional suburethral retropubic sling procedures; MUS: Mid-urethral sling; Retro-MUS: Retropubic mid-urethral sling; Transob-MUS: Transobturator mid-urethral sling; Single incision: Single incision sling procedures ('mini-slings')

For peer review only - http://bmjopen.bmj.com/site/about/guidelines.xhtml

Figure 2 Cost-effectiveness acceptability curves for the nine surgical treatments: lifetime time horizon

Sensitivity analysis (SA) and VOI analysis

In the first SA, results from the study by Keltie et al. (24) were used to inform the mesh complications incidence rates after Retro-MUS and Transob-MUS. Results from this SA show that Retro-MUS remains the most cost-effective option. However, when the incidence rate of mesh complications following Retro-MUS and Transob-MUS is increased to 10%, and then 20%, Trad-sling becomes the most cost-effective intervention (Table S6). Results from the second SA show that when a longer duration for persistent pain is assumed (3 and 5 years), the probability of Retro-MUS being the most cost-effective option decreases and the respective probabilities for Trad-sling and Open-colpo increase. Furthermore, results from the third SA show that when a higher incidence rate (10% and 20%) of persistent pain after Retro-MUS and Transob-MUS is assumed, the probability of Retro-MUS being the most cost-effective option decreases and the respective probability for Trad-sling increases. Detailed results from all sensitivity analyses are reported in Tables S6, S7, S8, S9, and S10, respectively.

The VOI analyses results show that the EVPI per patient is £11,857 and the estimated EVPI for the UK population for 1 year is £177.9 million. This figure increases as the time horizon (or period of time over which the information would be useful) is increased (Table 4). Results from the EVPPI analysis also show that the largest value appears to be in removing uncertainty around the incidence rate of complications and treatment effectiveness parameters. Results from this analysis are presented in Table S11.

Table 4 Results from Expected Value of Information Analysis

**The expected value of removing all current decision uncertainty**	**Overall EVPI (£)**	**Overall EVPI (QALY)**
Per person affected by the decision	11,857	0.56
Per year in UK assuming 15000 persons affected per year	177,855,000	8,385
Over 5 years	889,275,000	41,930
Over 10 years	1,778,550,000	83,850
Over 15 years	2,667,825,000	125,800
Over 20 years	3,557,100,000	167,700

Discussion

To our knowledge, this economic evaluation is the most comprehensive assessment of the cost-effectiveness of surgical interventions for the treatment of SUI or stress-predominant MUI in women. While the broader study upon which this paper is based (9) presents the entire spectrum of evidence available on the clinical effectiveness, safety and cost-effectiveness of surgical treatments for SUI, the work presented here focusses specifically on the economic viability of these treatments. Additionally, given that the cost estimates included in the previous study are now outdated, we have considered the relative cost-effectiveness of alternative interventions utilising cost information from the most recent price year (2019). Due to limited long-term clinical data, the results are uncertain but suggest that Retro-MUS is the least costly and most cost-effective surgical intervention over 10-year and lifetime time horizons; results which are largely driven by the low initial cost of Retro-MUS. This is primarily due to the fact that this procedure is conducted in a day-case setting, and there is a lower chance of needing repeat surgery due to its higher cure rate compared to all other surgical treatments (except for Trad-

For peer review only - http://bmjopen.bmj.com/site/about/guidelines.xhtml

sling). Trad-sling is the one alternative intervention, which has a relatively high probability of being cost-effective over 10-year and lifetime time horizons. This is likely driven by the high success rate associated with Trad-sling, relative to Retro-MUS. Over a 1–year time horizon, Retro-MUS is dominated (more costly and less effective) by Single incision sling, primarily due to the low initial cost of this procedure and the low complication rate associated with this type of surgery.

One of the strengths of our economic model is the evaluation of *nine* different surgical treatments in one study, informed by data from a comprehensive evidence synthesis and network meta-analysis where all the direct and indirect evidence (120 trials) were used to estimate the relative effectiveness of different surgical treatments in terms of cure rates (9). Very few cost-effectiveness studies in this clinical area have included complications within their analysis, despite the fact that the incidence of each complication can have an impact on the woman's quality of life, and result in costs incurred for the health system. Therefore, in the present study the impact that complications such as infection, urge urinary incontinence, voiding difficulties, bladder or urethral perforation, mesh removal, short-term pain and persistent pain have on costs and effects have been incorporated into the model and explored in sensitivity analysis. Approximately 50% of women who have SUI (4) also suffer from UUI symptoms; given that UUI potentially affects the woman's quality of life more than SUI (25), treatment pathways associated with UUI are also incorporated in the model to more accurately estimate QALYs for this patient group. The analysis was conducted in accordance with the ISPOR modelling guidelines (26) and extensive sensitivity analyses were undertaken to explore and characterise uncertainty in the model parameters. Our results were generally robust to the sensitivity analyses performed and are comparable to the findings of other published studies.

There are a number of published cost-effectiveness analyses evaluating some of the surgeries that we have assessed, the general findings of which are presented to allow for comparison. Two studies compared the single incision versus mid-urethral sling procedures over a 1-year time horizon, concluding that single incision was less costly and of similar effectiveness (27, 28). A UK study compared the cost-effectiveness of Retro-MUS (Tension-free Vaginal Tape (TVT)) versus open colposuspension, laparoscopic colposuspension, Trad-sling and injectable agents, concluding that TVT dominated Open-colpo over a 5-year time horizon (29). A cost–utility analysis in the UK to assess the cost-effectiveness of TVT compared with open Burch colposuspension found that TVT was less costly and more effective (20). A further study to assess the cost-effectiveness of TVT versus laparoscopic mesh colposuspension concluded that TVT was more cost-effective over a 1-year time horizon (30). Although there are limitations with many of these studies and heterogeneity present in the methods, the results from all of the above studies are largely in agreement with the findings from our economic model; generally supporting the conclusion that Retro-MUS (TVT) is likely to be the most cost-effective option when compared to the other types of surgeries for the treatment of SUI and stress-predominant MUI.

Results from the VOI analysis indicate that further research should focus on adverse events that, however rare, can have a detrimental impact on women's quality of life when they do occur (e.g. tape extrusion/exposure).

Limitations

One of the main limitations of the current study is the lack of long-term data which necessitated the extrapolation of relatively short-term data to 10 years, and over the lifetime, of women included in the analysis. Therefore, the results presented would only apply in a situation where relative differences in the effectiveness of Retro-MUS compared with the comparators do not change with longer follow-up. The long-term incidence of complications after the surgical treatments are also currently unknown. All of the estimated incidence rates for complications after each surgical treatment were based on data from trials with relatively short-term follow-up times. We tested the impact of possible higher incidence rates of some of the complications on the results, to incorporate and explore some of this uncertainty within our model. Finally, the economic analysis included costs to the NHS only. As highlighted earlier, individual patients also buy different products and incur the costs of containment products themselves; a future analysis may also need to consider these out-of-pocket costs to patients.

For peer review only - http://bmjopen.bmj.com/site/about/guidelines.xhtml

Conclusions

The results of this study suggest that, in the medium-and long-term, Retro-MUS is less costly and more effective than all other surgical interventions for the treatment of SUI; therefore, it is a dominant strategy. However, the results should be interpreted with caution as the long-term performance of all surgical treatments in terms of both continence and unanticipated adverse effects is not reliably known. Therefore, the results presented are based on the extrapolation of short- and medium-term evidence.

Acknowledgements

We would like to acknowledge all those involved in the wider study exploring the effectiveness and cost-effectiveness of surgical treatments for women with stress urinary incontinence (9).

For peer review only

References

1. Kwon BE, Kim GY, Son YJ, Roh YS, You MA. Quality of life of women with urinary incontinence: a systematic literature review. International neurourology journal. 2010;14(3):133-8.
2. Papanicolaou S, Pons ME, Hampel C, Monz B, Quail D, Schulenburg MG, et al. Medical resource utilisation and cost of care for women seeking treatment for urinary incontinence in an outpatient setting. Examples from three countries participating in the PURE study. Maturitas. 2005;52 Suppl 2:S35-47.
3. Hannestad YS, Rortveit G, Sandvik H, Hunskaar S. A community-based epidemiological survey of female urinary incontinence: the Norwegian EPINCONT study. Epidemiology of Incontinence in the County of Nord-Trondelag. J Clin Epidemiol. 2000;53(11):1150-7.
4. Hunskaar S, Lose G, Sykes D, Voss S. The prevalence of urinary incontinence in women in four European countries. BJU Int. 2004;93(3):324-30.
5. Turner DA, Shaw C, McGrother CW, Dallosso HM, Cooper NJ. The cost of clinically significant urinary storage symptoms for community dwelling adults in the UK. BJU Int. 2004;93(9):1246-52.
6. Urinary incontinence in women: the management of urinary incontinence in women CG171: National Institute for Health and Care Excellence 2013 [Available from: https://www.nice.org.uk/guidance/cg171/evidence/urinary-incontinence-in-women-full-guideline-191581165.
7. Sung VW, Schleinitz MD, Rardin CR, Ward RM, Myers DL. Comparison of Retropubic versus Transobturator Approach to Midurethral Slings: A Systematic Review and Meta-Analysis. Am J Obstet Gynecol. 2007;197(1):3-11.
8. Chuang K-W, Firoozi F. Surgical Management of Stress Urinary Incontinence. In: Firoozi F, editor. Female Pelvic Surgery. New York, NY: Springer New York; 2015. p. 39-54.
9. Brazzelli M, Javanbakht M, Imamura M, Hudson J, Moloney E, Becker F, et al. The Effectiveness and cost-effectiveness of Surgical Treatments for womEn with stRess urinary incontinence: An evidence synthesis, economic evaluation and discrete choice experiment (ESTER). 2017.
10. Morling J, Wood R. Using routinely available health data to examine the provision of, and outcomes following, surgery for SUI and POP in Scotland: Technical Report Information Services Division, NHS National Services Scotland; 2015 [Available from: http://www.gov.scot/Publications/2015/10/8485/downloads.

For peer review only - http://bmjopen.bmj.com/site/about/guidelines.xhtml

11. Excellence NIfHaC. Guide to the methods of technology appraisal 2013 2013 [Available from: https://www.nice.org.uk/process/pmg9/resources/guide-to-the-methods-of-technology-appraisal-2013-pdf-2007975843781.
12. TreeAge Pro, TreeAge Software, Williamstown, MA 2017 [Available from: https://www.treeage.com.
13. Cody J, Wyness L, Wallace S, Glazener C, Kilonzo M, Stearns S, et al. Systematic review of the clinical effectiveness and cost-effectiveness of tension-free vaginal tape for treatment of urinary stress incontinence. Health Technol Assess. 2003;7(21):iii, 1-189.
14. Lunn DJ, Thomas A, Best N, Spiegelhalter D. WinBUGS-a Bayesian modelling framework: concepts, structure, and extensibility. Statistics and computing. 2000;10(4):325-37.
15. Zilberman D, Kaplan S, Wesseler J. The loss from underutilizing GM technologies. AgBioForum. 2015;18(3):312-9.
16. Imamura M, Abrams P, Bain C, Buckley B, Cardozo L, Cody J, et al. Systematic review and economic modelling of the effectiveness and cost-effectiveness of non-surgical treatments for women with stress urinary incontinence. Health Technol Assess. 2010;14(40):1-188, iii-iv.
17. Health Do. Reference Costs 2017-18 2018 [Available from: https://improvement.nhs.uk/resources/reference-costs/.
18. Curtis L, Burns A. Unit Costs of Health & Social Care 2016: The University of Kent; 2016.
19. British National Formulary. BNF Online [Available from: https://www.medicinescomplete.com/mc/?utm_source=bnforg&utm_medium=homepage&utm_campaign=medicinescomplete.
20. Manca A, Sculpher MJ, Ward K, Hilton P. A cost-utility analysis of tension-free vaginal tape versus colposuspension for primary urodynamic stress incontinence. BJOG : an international journal of obstetrics and gynaecology. 2003;110(3):255-62.
21. Haywood KL, Garratt AM, Lall R, Smith JF, Lamb SE. EuroQol EQ-5D and condition-specific measures of health outcome in women with urinary incontinence: reliability, validity and responsiveness. Qual Life Res. 2008;17(3):475-83.
22. Kunkle CM, Hallock JL, Hu X, Blomquist J, Thung SF, Werner EF. Cost utility analysis of urethral bulking agents versus midurethral sling in stress urinary incontinence. Female pelvic med. 2015;21(3):154-9.
23. Shepherd JP, Lowder JL, Jones KA, Smith KJ. Retropubic and transobturator midurethral slings: a decision analysis to compare outcomes including efficacy and complications. Int Urogynecol J Pelvic Floor Dysfunct. 2010;21(7):787-93.
24. Keltie K, Elneil S, Monga A, Patrick H, Powell J, Campbell B, et al. Complications following vaginal mesh procedures for stress urinary incontinence: an 8 year study of 92,246 women. Scientific Reports. 2017;7(1):12015.
25. Tincello D, Sculpher M, Tunn R, Quail D, van der Vaart H, Falconer C, et al. Patient Characteristics Impacting Health State Index Scores, Measured by the EQ-5D of Females with Stress Urinary Incontinence Symptoms. Value Health. 2010;13(1):112-8.
26. Caro JJ, Briggs AH, Siebert U, Kuntz KM. Modeling good research practices--overview: a report of the ISPOR-SMDM Modeling Good Research Practices Task Force--1. Value Health. 2012;15(6):796-803.
27. Costantini E, Lazzeri M, Bini V, Zucchi A, Scarponi E, Porena M. Managing female urinary incontinence: a regional prospective analysis of cost-utility ratios (curs) and effectiveness. Archivio italiano di urologia, andrologia : organo ufficiale [di] Societa italiana di ecografia urologica e nefrologica. 2014;86(2):112-7.
28. Boyers D, Kilonzo M, Mostafa A, Abdel-Fattah M. Comparison of an adjustable anchored single-incision mini-sling, Ajust®, with a standard mid-urethral sling, TVT-OTM: a health economic evaluation. BJU Int. 2013;112(8):1169-77.
29. Kilonzo M, Vale L, Stearns SC, Grant A, Cody J, Glazener CM, et al. Cost effectiveness of tension-free vaginal tape for the surgical management of female stress incontinence. Int J Technol Assess Health Care. 2004;20(4):455-63.
30. Valpas A, Rissanen P, Kujansuu E, Nilsson CG. A cost-effectiveness analysis of tension-free vaginal tape versus laparoscopic mesh colposuspension for primary female stress incontinence. Acta Obstet Gynecol Scand. 2006;85(12):1485-90.

For peer review only - http://bmjopen.bmj.com/site/about/guidelines.xhtml

Footnotes

Funding statement: This research was commissioned by the NIHR HTA Programme as project number 15/09/06. The views expressed are those of the authors and not necessarily those of the NHS, the NIHR or the Department of Health and Social Care, UK. The funders were not actively involved in the research process at any stage. The study design, collection, analysis and interpretation of data, the writing of the manuscript and the decision to submit it for publication were all performed independent of the funders.

Competing interests statement: None declared.

Patient consent for publication: Not required.

Author contributions: MJ, DC, EM, LT and LV designed the study. MJ and EM built the model and ran the analyses. DC, MB, LS, PM, JH, GM and LV helped to inform the model. All co-authors (EM, MB, SW, LT, MIO, AM, LS, PM, FB, MI, JH, MS, GM, LV, DC) commented on the draft version of the manuscript and approved its final version.

Ethics approval and consent to participate: Not applicable.

Data availability statement: The authors declare that all of the data supporting the findings of this study are available within the article (or in the Supplementary Information).

For peer review only

For peer review only - http://bmjopen.bmj.com/site/about/guidelines.xhtml

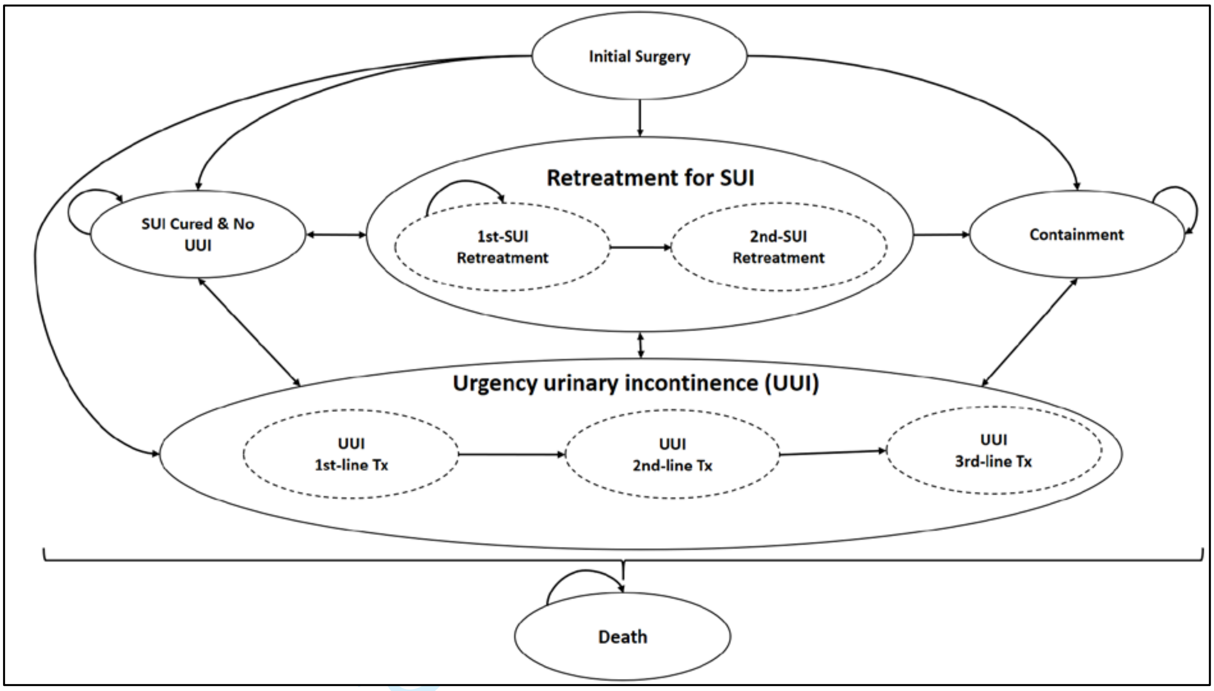

Figure 1 Markov model structure

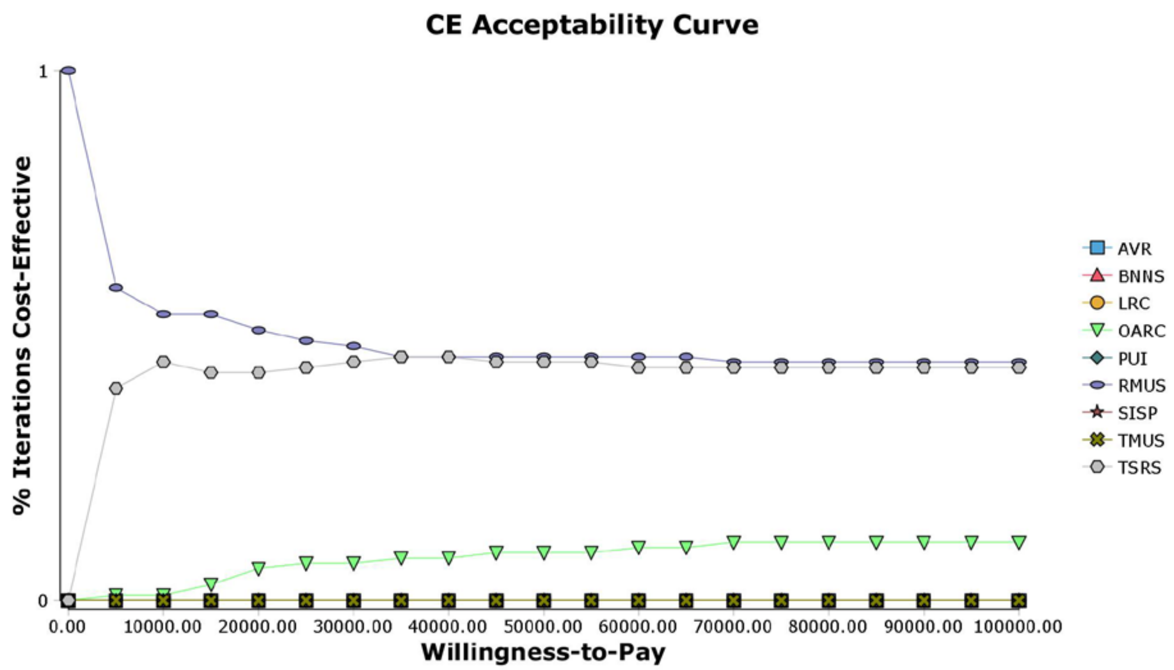

Figure 2 Cost-effectiveness acceptability curves for the nine surgical treatments: lifetime time horizon
Anterior repair: Anterior vaginal repair (anterior colporrhaphy); Bladder neck needle: Bladder neck needle suspensions; Lap-colpo: Laparoscopic retropubic colposuspension; Open-colpo: Open abdominal retropubic colposuspension; Trad-sling: Traditional suburethral retropubic sling procedures; MUS: Mid-urethral sling; Retro-MUS: Retropubic mid-urethral sling; Transob-MUS: Transobturator mid-urethral sling; Single incision: Single incision sling procedures ('mini-slings')

Appendices

Table S1 Long-term cure rates after Retro-MUS used for extrapolation

Scenario	Time (years)	Cure rate	Source	Scale parameter (λ)	Shape parameter (γ)
Base case analysis	1	84%	Meta-analysis	0.08799444	0.4932644
	2	78%			
Sensitivity analysis 1	1	85%	(1)	0.1969761	0.12368398
	2	80%			
Sensitivity analysis 2	1	78%	(2)	0.19391258	0.1788069
	13	67.5%			

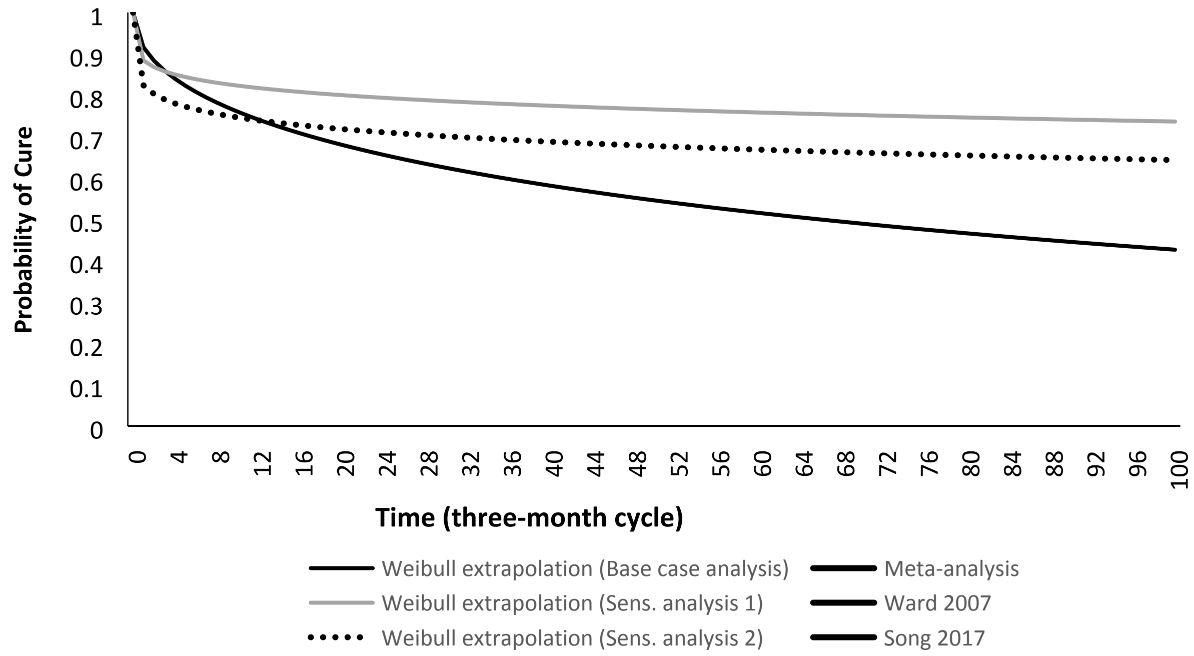

Figure S1 Extrapolated long-term cure rates after Retro-MUS surgery. Note: each cycle is 3 months long

For peer review only - http://bmjopen.bmj.com/site/about/guidelines.xhtml

Table S2 Weibull hazard function parameters for estimation of long-term cure rates after Injectable agents

**Weibull Parameters**	**Cure rate**		
	Lower limit	Mean	Upper limit
**Lambda**	1.020173	0.767078	0.548895
**Gamma**	0.120006	0.137820	0.166837

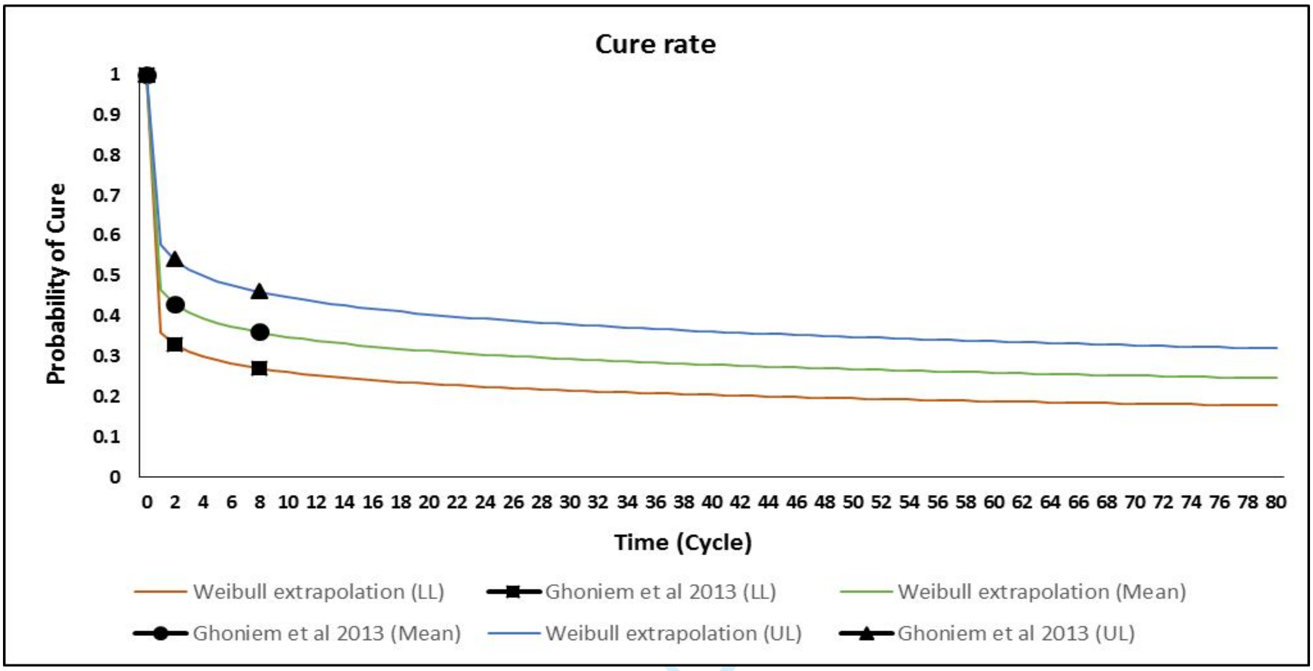

Figure S2 Extrapolated long-term cure rates after peri-urethral injections (Injectable agents). Note that each cycle is 3 months long

For peer review only - http://bmjopen.bmj.com/site/about/guidelines.xhtml

Table S3 Complications incidence rates after different surgical treatments (results from random-effect meta-analysis)

**Complication**	**Infection**		**De novo symptoms of urgency or urgency incontinence**		**Voiding difficulties including urinary retention**		**Bladder or urethral perforation**		**Tape/mesh erosion / extrusion/exposure**		**Short term pain**		**Persistent pain or discomfort**	
Surgery	Posterior Mean	Posterior SD	Posterior Mean	Posterior SD	Posterior Mean	Posterior SD	Posterior Mean	Posterior SD	Posterior Mean	Posterior SD	Posterior Mean	Posterior SD	Posterior Mean	Posterior SD
Anterior repair	NA	NA	15.84%	11.83%	0.01%	0.15%	NA	NA	NA	NA	NA	NA	NA	NA
Bladder neck needle	NA	NA	8.14%	4.87%	1.67%	2.61%	NA	NA	NA	NA	57.64%	30.08%	NA	NA
Open-colpo	24.33%	21.82%	5.78%	1.15%	3.00%	1.04%	1.02%	0.50%	0.0%	NA	20.94%	25.65%	0.15%	2.58%
Lap-colpo	NA	NA	8.38%	3.98%	6.32%	2.35%	2.10%	1.22%	0.0%	NA	NA	NA	NA	NA
Trad-sling	14.85%	12.49%	7.85%	3.23%	11.63%	3.60%	1.82%	1.11%	0.17%	0.34%	28.99%	28.12%	7.00%	8.98%
Retro-MUS	3.91%	4.72%	4.30%	0.66%	6.75%	0.95%	4.89%	0.50%	1.40%	0.31%	4.36%	1.14%	5.09%	2.37%
Transob-MUS	2.84%	1.03%	5.31%	0.51%	2.88%	0.44%	5.13%	0.50%	2.08%	0.26%	7.04%	2.08%	4.93%	1.22%
Single incision	3.22%	1.20%	6.47%	0.81%	2.15%	0.45%	0.37%	0.18%	2.38%	0.55%	4.54%	3.21%	1.28%	1.39%
Injectable agents	17.94%	24.59%	17.94%	24.59%	11.47%	20.70%	NA	NA	NA	NA	NA	NA	NA	NA

NA: Not available or applicable; Anterior repair: Anterior vaginal repair (anterior colporrhaphy); Bladder neck needle: Bladder neck needle suspensions; CrI: Creditable interval; Lap-colpo: Laparoscopic retropubic colposuspension; Open-colpo: Open abdominal retropubic colposuspension; Trad-sling: Traditional suburethral retropubic sling procedures; MUS: Mid-urethral sling; Retro-MUS: Retropubic mid-urethral sling; Transob-MUS: Transobturator mid-urethral sling; Single incision: Single incision sling procedures (‘mini-slings

For peer review only

For peer review only - http://bmjopen.bmj.com/site/about/guidelines.xhtml

Table S4 Costs inputs

Resource use	Cost (£)	Source	HRG code
**Surgery cost**			
Anterior repair	2,254.72	NHS reference costs 2017/18 (3)	MA04C/D - Intermediate open, lower genital tract procedures-elective inpatient
Bladder neck needle	1,978.94	NHS reference costs 2017/18 (3)	LB26A/B - Intermediate endoscopic, prostate or bladder neck procedures-elective
Open-colpo	4,113.30	NHS reference costs 2017/18 (3)	LB59Z - Major, open or laparoscopic bladder neck procedures-elective inpatient
Lap-colpo	4,113.30	NHS reference costs 2017/18 (3)	LB59Z - Major, open or laparoscopic bladder neck procedures-elective inpatient
Trad-sling	2,131.88	NHS reference costs 2017/18 (3)	LB51A/B - Vaginal tape operations for urinary incontinence-elective inpatient
Retro-MUS	1,550.29	NHS reference costs 2017/18 (3)	LB51A/B - Vaginal tape operations for urinary incontinence-day case
Transob-MUS	1,550.29	NHS reference costs 2017/18 (3)	LB51A/B - Vaginal tape operations for urinary incontinence-day case
Single incision	1,103.53	Boyers et al. 2013 (4)	N/A
Injectable agents	1,930.49	Cody et al. 2003 (5)	N/A
Additional costs associated with surgery			
Incontinence pads	112.68 (3 month cost estimate)	NICE guidance document (6)	N/A
Urodynamic test	164.38	NHS reference costs 2017/18 (3)	N/A
Consultation pre-surgery (20 minute consultation with surgical consultant)	48.78	PSSRU 2016 (7)	N/A
Urine dipstick analysis	4.18	NICE clinical guidelines (6)	N/A
Full blood count	6.69	NICE clinical guidelines (6)	N/A
Paracetamol 500mg (AAH) x 32	0.17 (daily cost based on a	BNF 2016 (8)	N/A
Lactulose 10g/15ml (AAH) x 10	0.53 (daily cost based on a	BNF 2016 (8)	N/A

For peer review only - http://bmjopen.bmj.com/site/about/guidelines.xhtml

**Urge incontinence treatment costs**			
Bladder training	103.73 (3 month cost estimate)	Appendices of NICE guidelines (6)	N/A
Band 6 hospital-based physiotherapist	47.72	PSSRU 2016 (7)	N/A
Oxybutynin 5mg (AAH) x 56	0.05 (daily cost based on a	BNF 2016 (8)	N/A
Consultant-led non-admitted follow-up	106.05	NHS reference costs 2017/18 (3)	N/A
Intermediate endoscopic bladder	1,029.78	NHS reference costs 2017/18 (3)	N/A
Botulinum toxin 100 unit powder for	146.57	BNF 2016 (8)	N/A
**Adverse event treatment costs**			
Co-amoxiclav 250/125mg (AAH) x 21	0.29 (daily cost based on a	BNF 2016 (8)	N/A
Attention to suprapubic bladder catheter	130.45	NHS reference costs 2017/18 (3)	N/A
Self-catheterisation	6.58 (daily cost based on 4	NICE guidance document (6)	N/A
Mesh excision or repair	1,396.57	NHS reference costs 2017/18 (3)	N/A
Aspirin 300mg (AAH) x 28	0.42 (daily cost based on a	BNF 2016 (8)	N/A

For peer review only - http://bmjopen.bmj.com/site/about/guidelines.xhtml

Table S5 Utility values for different health states and utility decrement associated with complications

**Health status**	**Value**	**Source**	**Duration**
SUI pre-treatment	0.78	(9)	Variable
Mixed pre-treatment	0.78	(9)	Variable
Cured from SUI, and no urge urinary incontinence	0.85	(10)	Variable
Urge urinary incontinence	0.74	(10)	Variable
Pure SUI re-treatment	0.74	(10)	Variable
Mixed re-treatment	0.74	(10)	Variable
SUI failure of treatment (containment)	0.74	(10)	Variable
Mixed failure of treatment (containment)	0.74	(10)	Variable
**Utility decrement values for adverse events**			
Adverse event	**Utility decrement**	**Source**	**Duration**
Infection	- 0.19	(11)	2 weeks
Voiding difficulties (long-term)	- 0.23	(11)	3 months
Bladder/urethral perforation	- 0.08	(11)	2 weeks
Tape/mesh exposure/erosion	- 0.25	(11)	3 months
Short-term pain	-0.25	(11)	2 weeks
Persistent pain	- 0.25	(11)	6 months

For peer review only - http://bmjopen.bmj.com/site/about/guidelines.xhtml

For peer review only

Deterministic Sensitivity Analyses Results

Table S6 Results from deterministic sensitivity analyses-applying higher incidence rates of mesh complications after MUS procedures (lifetime time horizon)

Strategy	Cost (£)	Incremental cost (£)	QALY	Incremental QALY	ICER (£) (ΔCost/ΔQALY)	Probability cost-effective for different WTP thresholds (%)	
						£20,000	£30,000
Base-case analysis – estimating the incidence rate of mesh complications after MUS procedures based on the data from trials							
Retro-MUS	8,666		24.005			51.0%	48.0%
Trad-sling	9,071	405	24.014	0.009	45,340	43.0%	45.0%
Transob-MUS	10,174	1,103	23.435	-0.580	Dominated	0%	0%
Single incision sling	10,189	1,118	23.221	-0.793	Dominated	0%	0%
Injectable agents	10,292	1,221	23.512	-0.503	Dominated	0%	0%
Bladder neck needle	10,803	1,732	23.312	-0.702	Dominated	0%	0%
Open-colpo	11,605	2,535	23.839	-0.175	Dominated	6.0%	7.0%
Anterior repair	11,609	2,539	23.168	-0.847	Dominated	0%	0%
Lap-colpo	12,440	3,369	23.522	-0.492	Dominated	0%	0%
SA – estimating the incidence rate of mesh complications for Retropubic MUS (3.7%) and Transobturator MUS (2.8%) based on the data from Keltie and colleagues							
Retro-MUS	8,702		24.094			50.0%	50.0%
Trad-sling	9,087	385	24.094	-0.000	Dominated	47.0%	46.0%
Transob-MUS	10,208	1,506	23.511	-0.583	Dominated	0%	0%
Single incision sling	10,210	1,508	23.315	-0.779	Dominated	0%	0%
Injectable agents	10,278	1,576	23.550	-0.544	Dominated	0%	0%
Bladder neck needle	10,814	2,112	23.385	-0.710	Dominated	0%	0%
Anterior repair	11,605	2,903	23.211	-0.883	Dominated	0%	0%
Open-colpo	11,618	2,916	23.905	-0.189	Dominated	2.0%	3.0%
Lap-colpo	12,439	3,737	23.629	-0.465	Dominated	1.0%	1.0%
SA –assuming 10% incidence rate of mesh complications after Retropubic MUS and Transobturator MUS							
Retro-MUS	8,796		23.976			47.0%	45.0%
Trad-sling	9,093	297	23.988	0.013	23,783	50.0%	50.0%
Single incision sling	10,201	1,107	23.170	-0.818	Dominated	0%	0%
Injectable agents	10,293	1,200	23.490	-0.498	Dominated	0%	0%

For peer review only - http://bmjopen.bmj.com/site/about/guidelines.xhtml

Transob-MUS	10,295	1,202	23.370	-0.618	Dominated	0%	0%
Bladder neck needle	10,804	1,711	23.307	-0.681	Dominated	0%	0%
Anterior repair	11,606	2,513	23.122	-0.866	Dominated	0%	0%
Open-colpo	11,618	2,524	23.826	-0.162	Dominated	3.0%	5.0%
Lap-colpo	12,438	3,345	23.491	-0.497	Dominated	0%	0%
SA –assuming 20% incidence rate of mesh complications after Retropubic MUS and Transobturator MUS							
Retro-MUS	8,906		24.021			42.0%	40.0%
Trad-sling	9,098	192	24.090	0.069	2,777	50.0%	49.0%
Single incision sling	10,200	1,102	23.317	-0.773	Dominated	0%	0%
Injectable agents	10,284	1,186	23.572	-0.519	Dominated	0%	0%
Transob-MUS	10,415	1,317	23.458	-0.633	Dominated	0%	0%
Bladder neck needle	10,806	1,708	23.417	-0.673	Dominated	0%	0%
Anterior repair	11,617	2,519	23.258	-0.833	Dominated	0%	0%
Open-colpo	11,623	2,525	23.944	-0.147	Dominated	8.0%	11.0%
Lap-colpo	12,440	3,342	23.653	-0.437	Dominated	0%	0%

Table S7 Results from deterministic sensitivity analyses-incorporating longer duration for persistent pain complication (lifetime time horizon)

Strategy	Cost (£)	Incremental cost (£)	QALY	Incremental QALY	ICER (£) (ΔCost/ΔQALY)	Probability cost-effective for different WTP thresholds (%)	
						**£20,000**	**£30,000**
Base-case analysis- assuming that persistent pain complication will last on average for 6 months							
Retro-MUS	8,666		24.005			51.0%	48.0%
Trad-sling	9,071	405	24.014	0.009	45,340	43.0%	45.0%
Transob-MUS	10,174	1,103	23.435	-0.580	Dominated	0%	0%
Single incision sling	10,189	1,118	23.221	-0.793	Dominated	0%	0%
Injectable agents	10,292	1,221	23.512	-0.503	Dominated	0%	0%
Bladder neck needle	10,803	1,732	23.312	-0.702	Dominated	0%	0%
Open-colpo	11,605	2,535	23.839	-0.175	Dominated	6.0%	7.0%
Anterior repair	11,609	2,539	23.168	-0.847	Dominated	0%	0%
Lap-colpo	12,440	3,369	23.522	-0.492	Dominated	0%	0%
SA – assuming that persistent pain complication will last on average for 36 months							
Retro-MUS	8,662		23.902			39.0%	35.0%

For peer review only - http://bmjopen.bmj.com/site/about/guidelines.xhtml

Trad-sling	9,097	435	23.911	0.009	30,466	47.0%	42.0%
Transob-MUS	10,174	1,077	23.304	-0.607	Dominated	0%	0%
Single incision sling	10,194	1,097	23.182	-0.729	Dominated	0%	0%
Injectable agents	10,299	1,202	23.533	-0.378	Dominated	0%	0%
Bladder neck needle	10,814	1,717	23.341	-0.570	Dominated	0%	0%
Open-colpo	11,593	2,496	23.842	-0.069	Dominated	12.0%	20.0%
Anterior repair	11,607	2,510	23.122	-0.789	Dominated	0%	0%
Lap-colpo	12,437	3,340	23.564	-0.347	Dominated	2.0%	3.0%
SA – assuming that persistent pain complication will last on average for 60 months							
Retro-MUS	8,677		23.781			51.0%	41.0%
Traditional sling	9,075	397	23.721	-0.060	Dominated	25.0%	24.0%
Single incision sling	10,186	1,509	23.162	-0.619	Dominated	0%	0%
Transob-MUS	10,187	1,509	23.201	-0.580	Dominated	0%	0%
Injectable agents	10,283	1,606	23.474	-0.307	Dominated	2.0%	2.0%
Bladder neck needle	10,806	2,129	23.303	-0.478	Dominated	0%	0%
Anterior repair	11,606	2,928	23.153	-0.628	Dominated	0%	0%
Open-colpo	11,614	2,937	23.843	0.062	53,948	22.0%	33.0%
Lap-colpo	12,432	818	23.574	-0.256	Dominated	0%	0%

Table S8 Results from deterministic sensitivity analyses-incorporating higher incidence rate for persistent pain complication after MUS procedures (lifetime time horizon)

Strategy	Cost (£)	Incremental cost (£)	QALY	Incremental QALY	ICER (£) (ΔCost/ΔQALY)	Probability cost-effective for different WTP thresholds (%)	
						£20,000	£30,000
Base-case analysis- assuming that incidence rate of persistent pain after Retropubic MUS and Transobturator MUS are 5.09% and 4.93%							
Retro-MUS	8,666		24.005			51.0%	48.0%
Trad-sling	9,071	405	24.014	0.009	45,340	43.0%	45.0%
Transob-MUS	10,174	1,103	23.435	-0.580	Dominated	0%	0%
Single incision sling	10,189	1,118	23.221	-0.793	Dominated	0%	0%
Injectable agents	10,292	1,221	23.512	-0.503	Dominated	0%	0%
Bladder neck needle suspensions	10,803	1,732	23.312	-0.702	Dominated	0%	0%
Open-colpo	11,605	2,535	23.839	-0.175	Dominated	6.0%	7.0%
Anterior repair	11,609	2,539	23.168	-0.847	Dominated	0%	0%

For peer review only - http://bmjopen.bmj.com/site/about/guidelines.xhtml

Lap-colpo	12,440	3,369	23.522	-0.492	Dominated	0%	0%
SA – assuming that incidence rate of persistent pain after Retropubic MUS and Transobturator MUS is 10%							
Retro-MUS	8,695		24.072			40.0%	39.0%
Trad-sling	9,089	394	24.152	0.080	4,730	57.0%	56.0%
Transob-MUS	10,916	1,107	23.533	-0.619	Dominated	0%	0%
Single incision sling	10,201	1,112	23.531	-0.621	Dominated	0%	0%
Injectable agents	10,284	1,195	23.601	-0.551	Dominated	0%	0%
Bladder neck needle suspensions	10,812	1,723	23.471	-0.681	Dominated	0%	0%
Anterior repair	11,610	2,521	23.274	-0.878	Dominated	0%	0%
Open-colpo	11,620	2,531	23.972	-0.180	Dominated	3.0%	5.0%
Lap-colpo	12,455	3,366	23.673	-0.479	Dominated	0%	0%
SA – assuming that incidence rate of persistent pain after Retropubic MUS and Transobturator MUS is 20%							
Retro-MUS	8,667		23.973			27.0%	26.0%
Trad-sling	9,079	412	24.084	0.111	3,590	64.0%	63.0%
Single incision sling	10,184	1,104	23.274	-0.810	Dominated	0%	0%
Transob-MUS	10,184	1,105	23.432	-0.652	Dominated	0%	0%
Injectable agents	10,261	1,182	23.553	-0.531	Dominated	0%	0%
Bladder neck needle suspensions	10,802	1,723	23.342	-0.742	Dominated	0%	0%
Anterior repair	10,597	2,518	23.191	-0.893	Dominated	0%	0%
Open-colpo	11,600	2,521	23.871	-0.213	Dominated	9.0%	11.0%
Lap-colpo	12,420	3,341	23.562	-0.522	Dominated	0%	0%

Table S9 Results from deterministic sensitivity analyses- applying higher incidence rates and longer duration for persistent pain after MUS procedures (lifetime time horizon)

Strategy	Cost (£)	Incremental cost (£)	QALY	Incremental QALY	ICER (£) (ΔCost/ΔQALY)	Probability cost-effective for different WTP thresholds (%)	
						£20,000	£30,000
Base-case analysis- assuming that incidence rate of persistent pain after Retropubic MUS and Transobturator MUS are 5.09% and 4.93% and average duration of persistent pain is 6 months							
Retro-MUS	8,666		24.005			51.0%	48.0%
Trad-sling	9,071	405	24.014	0.009	45,340	43.0%	45.0%
Injectable agents	10,174	1,103	23.435	-0.580	Dominated	0%	0%
Single incision sling	10,189	1,118	23.221	-0.793	Dominated	0%	0%

For peer review only - http://bmjopen.bmj.com/site/about/guidelines.xhtml

Transob-MUS	10,292	1,221	23.512	-0.503	Dominated	0%	0%
Bladder neck needle	10,803	1,732	23.312	-0.702	Dominated	0%	0%
Open-colpo	11,605	2,535	23.839	-0.175	Dominated	6.0%	7.0%
Anterior repair	11,609	2,539	23.168	-0.847	Dominated	0%	0%
Lap-colpo	12,440	3,369	23.522	-0.492	Dominated	0%	0%
SA – assuming that incidence rate of persistent pain after Retropubic MUS and Transobturator MUS are 20% and average duration of persistent pain is 60 months							
Retro-MUS	8,688		23.131			0%	0%
Trad-sling	9,079	391	23.752	0.621	630	46.0%	45.0%
Single incision sling	10,186	1,107	23.181	-0.571	Dominated	1.0%	1.0%
Transob-MUS	10,198	1,119	22.523	-1.229	Dominated	0%	0%
Injectable agents	10,301	1,222	23.511	-0.241	Dominated	11.0%	10.0%
Bladder neck needle	10,817	1,738	23.341	-0.411	Dominated	0%	0%
Open-colpo	11,599	2,520	23.834	0.082	30,319	39.0%	39.0%
Anterior repair	11,607	8	23.153	-0.681	Dominated	0%	0%
Lap-colpo	12,432	833	23.552	-0.282	Dominated	3.0%	5.0%

Table S10 Results from deterministic sensitivity analyses-applying different values for short and long term cure rates after retropubic MUS (lifetime time horizon)

Strategy	Cost (£)	Incremental cost (£)	QALY	Incremental QALY	ICER (£) (ΔCost/ΔQALY)	Probability cost-effective for different	
						£20,000	£30,000
Base-case analysis – applying estimated mean values from the meta-analysis for short and long-term cure rates after Retropubic MUS							
Retro-MUS	8,666		24.005			51.0%	48.0%
Trad-sling	9,071	405	24.014	0.009	45,340	43.0%	45.0%
Transob-MUS	10,174	1,103	23.435	-0.580	Dominated	0%	0%
Single incision sling	10,189	1,118	23.221	-0.793	Dominated	0%	0%
Injectable agents	10,292	1,221	23.512	-0.503	Dominated	0%	0%
Bladder neck needle	10,803	1,732	23.312	-0.702	Dominated	0%	0%
Open-colpo	11,605	2,535	23.839	-0.175	Dominated	6.0%	7.0%
Anterior repair	11,609	2,539	23.168	-0.847	Dominated	0%	0%
Lap-colpo	12,440	3,369	23.522	-0.492	Dominated	0%	0%
SA – applying values from Ward and colleagues,(1) for short and long-term cure rates after Retropubic MUS							

For peer review only

Retro-MUS	7,848		24.123			97.0%	95.0%
Trad-sling	9,195	1,347	23.682	-0.441	Dominated	1.0%	2.0%
Transob-MUS	10,111	2,263	23.222	-0.901	Dominated	0%	0%
Single incision sling	10,176	2,328	23.161	-0.962	Dominated	0%	0%
Injectable agents	10,290	2,442	23.484	-0.639	Dominated	0%	0%
Bladder neck needle	10,831	2,983	23.304	-0.819	Dominated	0%	0%
Anterior repair	11,599	3,751	23.131	-0.992	Dominated	0%	0%
Open-colpo	11,761	3,914	23.751	-0.372	Dominated	2.0%	3.0%
Lap-colpo	12,467	4,619	23.513	-0.610	Dominated	0%	0%
SA – applying data from Song and colleagues,(2) for short and long-term cure rates after Retropubic MUS							
Retro-MUS	8,107		24.014			91.0%	87.0%
Trad-sling	9,235	1,128	23.674	-0.340	Dominated	4.0%	4.0%
Transob-MUS	10,160	2,053	23.182	-0.832	Dominated	0%	0%
Single incision sling	10,204	2,097	23.143	-0.872	Dominated	0%	0%
Injectable agents	10,288	2,181	23.481	-0.533	Dominated	0%	0%
Bladder neck needle	10,830	2,722	23.291	-0.723	Dominated	0%	0%
Anterior repair	10,611	3,504	23.122	-0.892	Dominated	0%	0%
Open-colpo	11,794	3,686	23.731	-0.283	Dominated	4.0%	8.0%
Lap-colpo	12,494	4,387	23.513	-0.501	Dominated	1.0%	1.0%

For peer review only

Value of Information Analysis Results

Table S11 Results from Expected Value of Partial Perfect Information Analysis

Parameters	Per person EVPPI per year (£)	EVPPI for UK per year (£)
Health utility values	168	2,520,000
Operation related mortality rates	60	900,000
Relative treatment effectiveness	1,395	20,925,000
Combinations of parameters associated with all the complications	3,973	59,595,000
Combinations of parameters associated with short-term and persistent pain	430	6,450,000
Combinations of parameters associated with short-term and persistent pain and mesh erosion/removal	1,873	28,095,000

For peer review only

References

1. Ward KL, Hilton P. Tension-free vaginal tape versus colposuspension for primary urodynamic stress incontinence: 5-year follow up. BJOG : an international journal of obstetrics and gynaecology. 2008;115(2):226-33.
2. Song PH, Kwon DH, Ko YH, Jung HC. The Long-Term Outcomes of the Tension-free Vaginal Tape Procedure for Treatment of Female Stress Urinary Incontinence: Data from Minimum 13 Years of Follow-Up. LUTS: Lower Urinary Tract Symptoms. 2017;9(1):10-4.
3. Department of Health. Reference Costs 2017-182018.
4. Boyers D, Kilonzo M, Mostafa A, Abdel-Fattah M. Comparison of an adjustable anchored single-incision mini-sling, Ajust®, with a standard mid-urethral sling, TVT-OTM: a health economic evaluation. BJU Int. 2013;112(8):1169-77.
5. Cody J. Systematic review of the clinical effectiveness and cost-effectiveness of tension-free vaginal tape for treatment of urinary stress incontinence. 2003.
6. Urinary incontinence in women: management Clinical guideline [CG171] 2015 [Available from: https://www.nice.org.uk/guidance/cg171.
7. Curtis L, Burns A. Unit Costs of Health & Social Care 2016: The University of Kent; 2016.
8. British National Formulary. BNF Online [Available from: https://www.medicinescomplete.com/mc/?utm_source=bnforg&utm_medium=homepage&utm_campaign=medicinescomplete.
9. Manca A, Sculpher MJ, Ward K, Hilton P. A cost-utility analysis of tension-free vaginal tape versus colposuspension for primary urodynamic stress incontinence. BJOG : an international journal of obstetrics and gynaecology. 2003;110(3):255-62.
10. Haywood KL, Garratt AM, Lall R, Smith JF, Lamb SE. EuroQol EQ-5D and condition-specific measures of health outcome in women with urinary incontinence: reliability, validity and responsiveness. Qual Life Res. 2008;17(3):475-83.
11. Shepherd JP, Lowder JL, Jones KA, Smith KJ. Retropubic and transobturator midurethral slings: a decision analysis to compare outcomes including efficacy and complications. Int Urogynecol J Pelvic Floor Dysfunct. 2010;21(7):787-93.

For peer review only - http://bmjopen.bmj.com/site/about/guidelines.xhtml
